# High‐Entropy Ferroelectric‐Ferroelastic Hybrid for Ultrahigh and Temperature‐Insensitive Dielectric Energy Storage

**DOI:** 10.1002/advs.202518725

**Published:** 2025-11-17

**Authors:** Xuefan Zhou, He Qi, Yingchun Su, Zhimin Huang, Yan Zhang, Hang Luo, Dou Zhang

**Affiliations:** ^1^ Powder Metallurgy Research Institute State Key Laboratory of Powder Metallurgy Central South University Changsha Hunan 410083 China; ^2^ School of Materials Science and Technology Hainan University Haikou Hainan 570228 China

**Keywords:** energy storage, ferroelastics, high‐entropy, polar nanoregions, temperature stability

## Abstract

Electrostatic energy storage plays an irreplaceable role in the pulse power systems, thus the development of capacitors with ultrahigh and thermal stable energy storage properties meets the requirement of next‐generation devices. In this study, a high‐entropy ferroelectric‐ferroelastic hybrid perovskite material is successfully developed, in which the ultralow tolerance factor triggers the ordered oxygen octahedral tilting while high‐entropy effect breaks the polarization ordering. As a result, a unique hybrid architecture, ferroelastic microdomains spanning hundreds of nanometers embedded with randomly dispersed polar nanoregions of 1–3 nm, endows the ceramic with polar heterogeneity as well as lowered polarization hysteresis, delayed polarization saturation and enhanced breakdown strength owing to the large extra local elastic field from large ferroelastic distortion. Together with the thermal stable feature of ferroelastic domains, superior energy storage properties (the recoverable energy density of 15.35 J cm^−3^ and efficiency of 90.3% @ 70 kV mm^−1^) along with outstanding thermal stability over a wide temperature range of ‐40–200 °C can be detected based on the super‐stable dielectric response with a dielectric constant of 676 and temperature coefficient of capacitance ≤ ±15% over –100–320 °C. This work opens up not only a novel field of high‐entropy ferroelastics but also new possibilities for broadening the temperature stability range of dielectric energy storage materials.

## Graphical abstract

A high‐entropy ferroelectric‐ferroelastic hybrid perovskite material is successfully developed, in which a unique hybrid architecture, ferroelastic microdomainsembedded with randomly dispersed polar nanoregions, endows the ceramic with polar heterogeneity as well as lowered polarization hysteresis, delayed saturation polarization and enhanced breakdown strength.

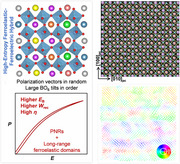

## 1 Introduction

**Figure 1:**
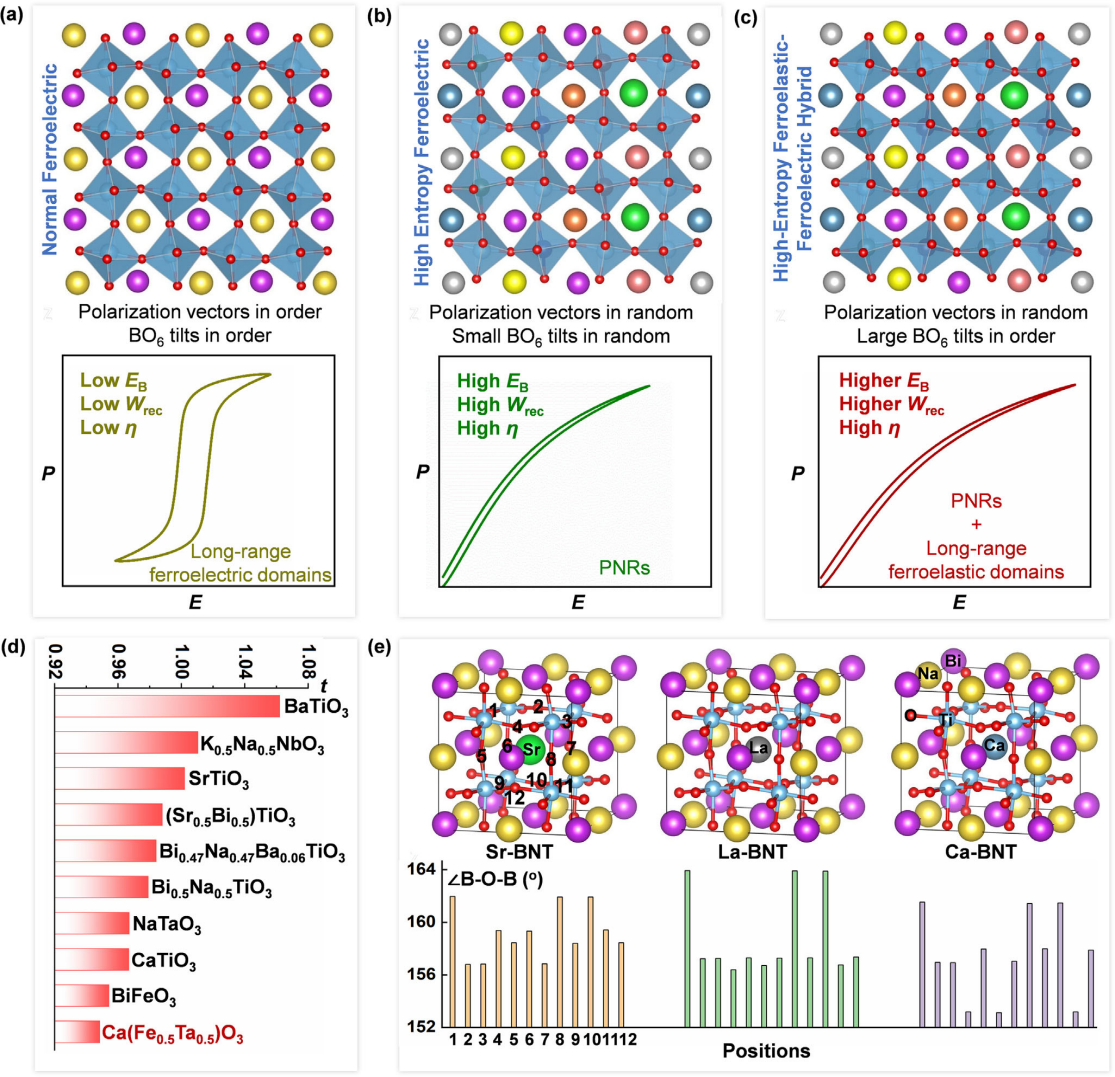
Schematics diagram of ferroelastic‐ferroelectric hybrid modulation in the high‐entropy perovskite oxides. Atomic configurations and *P*‐*E* loops of a) normal ferroelectric, b) high‐entropy ferroelectric, and c) high‐entropy ferroelastic‐ferroelectric hybrid. d) Tolerance factors of typical perovskite oxides, and the complex perovskite Ca(Fe_0.5_Ta_0.5_)O_3_ shows a low factor of 0.9485. e) DFT calculation: 2×2×2 supercells of Sr‐doped BNT, La‐doped BNT, and Ca‐doped BNT with *R*3c symmetry after structural relaxation, and variation of the degrees of ∠B‐O‐B (labeled 1 to 12 in the figure) indicates the key effect of Ca to strengthen oxygen octahedral tilting.

Dielectric capacitors exhibit ultrahigh power density and ultrafast charge‐discharge rates, playing an indispensable role in pulsed power technologies. Currently, BOPP polymer dielectrics represent the state‐of‐the‐art in commercial high‐energy‐density capacitors.^[^ ^1^ ^]^ They exhibit an exceptionally high breakdown strength (BDS) of up to 600 MV m^−1^, delivering a recoverable energy density (*W*_rec_) of 3–5 J cm^−^^3^ and an efficiency (*η*) exceeding 95 % at room temperature. However, the relatively low dielectric constant of BOPP (*ε*_r_ ≈ 2.2–2.5) and limited high‐temperature endurance (< 100 °C) have motivated extensive research into dielectric ceramic‐based capacitors for achieving higher energy density and improved thermal stability. A representative study involves an ultrahigh capacitive energy storage performance realized through a dendritic nanopolar design in a PbZr_0.53_Ti_0.47_O_3_–MgO film, which demonstrates a remarkable energy density of 215.8 J cm^−3^ with an efficiency of 80.7% under an electric field of 740 kV mm^−1^.^[^ ^2^ ^]^ Nevertheless, while several ceramic systems show promise at the laboratory scale, their path to widespread industrialization, even on a small scale, is hindered by significant challenges. The primary barriers are the high manufacturing costs, complexity associated with thinning, multilayering and flexibilization, and unresolved reliability concerns. Current research is shifting from solely pursuing high energy density toward optimizing comprehensive performance, including high *ε*_r_, low dielectric loss (tan*δ*), large *W*_rec_, and high *η* in ceramic dielectrics, while enhancing the stability and reliability of these key parameters.^[^ ^3^ ^]^ Stability and reliability are essential to withstand extreme operating conditions, including temperature extremes, high frequencies, and high electric fields. Compared with traditional normal ferroelectrics with long‐range ferroelectric domains (**Figure** **1**a), ergodic relaxor ferroelectrics (ERFE)^[^ ^4^, ^5^, ^6^, ^7^ ^]^ and superparaelectrics (SPE)^[^ ^8^, ^9^, ^10^, ^11^ ^]^ have demonstrated significant potential for optimizing *W*_rec_, *η*, stability and reliability.

In perovskite‐structured oxides (ABO_3_), multi‐element doping at both A‐ and B‐sites can enhance configurational entropy or even achieve high‐entropy effect (Figure 1b).^[^ ^4^, ^7^ ^]^ Disordered component distributions act to generate mismatched atomic radii, valence states, and electronegativities, and enhance the random local strain and electric fields, thereby disrupting long‐range ferroelectric order. Consequently, the *T*_f_ (dipole freezing temperature) is significantly suppressed. When *T*_f_ falls below room temperature, the material exhibits weakly coupled polar nanoregions (PNRs) at room temperature, establishing an ergodic relaxor ferroelectric (ERFE) or superparaelectric (SPE) state. Concurrently, compositional fluctuations drive heterogeneous lattice distortions, therefore, the ERFE or SPE state is usually accompanied by a polymorphic phase structure in nanoscale.^[^ ^4^, ^12^, ^13^, ^14^ ^]^ Here, SPE represents an ergodic relaxor state ranging from *T*_m_ (the temperature corresponding to the dielectric maximum) to *T*_B_ (Burns temperature).^[^ ^10^ ^]^ Compared to ERFE, the domains in SPE further scale down into highly dynamic PNRs with weakened interactions, benefiting the minimal polarization hysteresis and higher *η*. Besides, a higher driving electric field is required to achieve *P*_max_ in SPE, thereby maximizing the *W*_rec_.

Bi_0.5_Na_0.5_TiO_3_ system is attractive to develop high‐entropy SPE ceramics with superior and stable dielectric energy storage performance because of the high saturation polarization (≈50 µC cm^−^^2^) and unique double dielectric peaks with a broad dielectric platform.^[^ ^15^ ^]^ There have been many related high‐performance ceramic systems, such as 1/3Bi_0.5_Na_0.5_TiO_3_‐1/3BaTiO_3_‐1/3NaNbO_3_ (*W*_rec_ of 10.59 J cm^−3^, *η* of 87.6 % at 55 kV mm^−1^),^[^ ^9^ ^]^ (Bi_1/3_Ba_1/3_Na_1/3_)(Fe_2/9_Ti_5/9_Nb_2/9_)O_3_ (*W*_rec_ of 15.9 J cm^−3^, *η* of 93 % at 67 kV mm^−1^),^[^ ^16^ ^]^ and 0.658(Bi_0.47_Na_0.47_Yb_0.03_Tm_0.01_)TiO_3_‐0.0564Sr(Sn_0.5_Hf_0.5_)O_3_‐0.3(Ba_0.5_Sr_0.5_)TiO_3_ (*W*_rec_ of 11.23 J cm^−3^, *η* of 90.87 % at 67 kV mm^−1^),^[^ ^17^ ^]^ *etc*. Intriguingly, even among BNT‐based high‐entropy SPEs, significant variations in energy storage performance exist. This discrepancy stems primarily from the high compositional diversity and the lack of general guidelines. Different doping elements induce distinct local lattice distortions, including cation displacement and oxygen octahedral tilting. These distortions subsequently influence the phase structure, the scale and interaction of PNRs, the strength of spontaneous polarization, and even the breakdown strength, ultimately leading to divergent dielectric energy storage properties. However, current research on Bi_0.5_Na_0.5_TiO_3_‐based dielectric energy storage ceramics is strongly biased toward modulating cation displacement and enhancing local random fields by introducing multiple elements on equivalent sites,^[^ ^6^, ^13^, ^16^, ^18^ ^]^ with limited efforts dedicated to improving the degree and ordering of oxygen octahedral tilting.

Oxygen octahedral tilting usually serves as an antiferrodistortive distortion and also plays an important role in modulating the polarization response.^[^ ^19^, ^20^, ^21^, ^22^ ^]^ In ABO_3_ ferroelectric materials, polarization switching requires lattice atoms to overcome an energy barrier as they transition from one stable polarization direction to the other. Pronounced octahedral tilting modes couple with the original lattice symmetry, resulting in a more stable, lower‐symmetry phase. This reduced symmetry imposes stronger constraints on the possible displacement directions of cations. Furthermore, when an external electric field drives polarization reversal, it must not only induce cation displacement but also synchronously drive the rotation of the entire oxygen octahedral network—that is, a change in the tilting angle. The latter process involves the motion of oxygen ions, making it a slow and energy‐intensive step. The combined effect of these two mechanisms retards the polarization response, thus delays the polarization saturation and enhances the electrical breakdown strength. Consequently, the introduction of ordered oxygen octahedral tilting in high‐entropy ferroelectrics would be benefit for improving dielectric energy storage performance with high *W*_rec_ and high *η* under a high applying electric field (Figure 1c).

In this work, high‐entropy compositions (Bi_0.47_Na_0.47_Ba_0.06_)_1‐_ *_x_* Ca*_x_* Ti_1‐_ *_x_* Fe_0.5_ *_x_* Ta_0.5_ *_x_* O_3_ (BNBCTFT*_x_*) are specially designed to construct a hybrid between ferroelastic and relaxor ferroelectric. By regulating the relative amount of heterovalent cations on equivalent sites, the ideal configurational entropy (*S*_config_) apparently increased from 0.878R for *x* = 0 to 1.892R for *x* = 0.22, which has fallen into the high‐entropy region (Figure S1, Supporting Information). Here, high entropy strategy is adopted to construct the SPE state with atomic‐scale polar heterogeneity of dipoles. Besides, the perovskite tolerance factor (*t*) is the main rule guiding the selection of multiple elements to induce the long‐range ferroelastic distortion generated by oxygen octahedral tilting order (Figure 1d) and the key element Ca with small ion radius (1.34 Å) is the core to induce the substantial oxygen octahedral tilting and achieve long‐range order (Figure 1e). As expected, the high‐entropy ferroelectric‐ferroelastic is created to show a unique hybrid microstructure: long‐range ordered ferroelastic microdomains embedded with randomly scattered 1–3 nm PNRs, which endows the capacitor with superior dielectric energy storage performance and exceptional temperature stability.

## 2 Results and Discussion

### 2.1 Phase Structure Modulation

**Figure 2:**
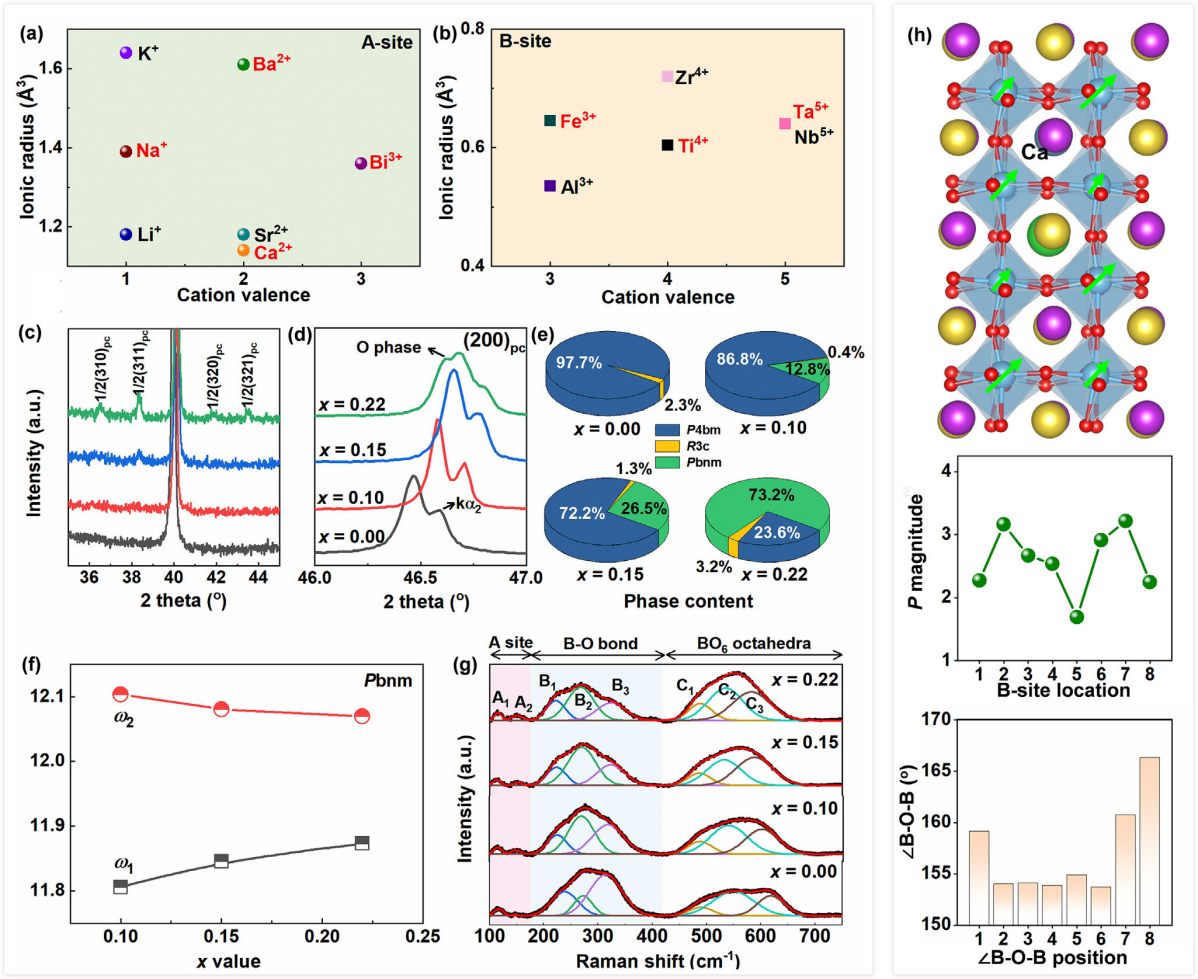
Crystal structure of BNBCTFT*_x_* ceramics. a,b) Distribution of ionic radii and valence states of A‐site and B‐site cations commonly used in BNT‐based solid solutions. c,d) XRD patterns in the 2*θ* range of 35‐45° and 46‐47° for the BNBCTFT*_x_* ceramics. e) Phase contents in the BNBCTFT*_x_* ceramics. f) oxygen octahedral tilting degrees in the *P*bnm phase with the increase of *x*. g) Room‐temperature Raman spectra of the BNBCTFT*_x_* ceramics. h) DFT calculation: 2×2×4 BNBT‐Ca supercell with *R*3c symmetry after structural relaxation, distribution of B‐site atom polarization vectors, magnitude of polarization vector and ∠B‐O‐B at different positions.

In the high‐entropy BNBCTFT*_x_* system, we choose Ca^2+^ (*r*≈1.14 Å) with a smaller ionic radius to substitute the Bi^3+^ (*r*≈1.36 Å), Na^+^ (*r*≈1.39 Å) and Ba^2+^ (*r*≈1.61 Å) at A‐site (**Figure** **2**a), and heterovalent ions Fe^3+^ (*r*≈0.645 Å) and Ta^4+^ (*r*≈0.64 Å) with larger ionic radii to replace Ti^4+^ (*r*≈0.605 Å) at B‐site (Figure 2b). As a result, the *t* value decreases from 0.984 to 0.976 as *x* increasing from 0 to 0.22, implying an increase in the driving force for local distortion of oxygen sublattice through oxygen octahedral tilting. In BNT‐based systems, the rhombohedral (*R*3c) and tetragonal (*P*4bm) structures correspond to the *a* ^−^ *a* ^−^ *a* ^−^ and *a* ^0^ *a* ^0^ *c* ^+^ oxygen octahedral tilting, respectively, and can give rise to the ½{*ooo*}and ½{*ooe*} superlattice reflections (“*o*” and “*e*” are odd and even Miller indices).^[^ ^23^, ^24^, ^25^ ^]^ Besides, the complex perovskite Ca(Fe_0.5_Ta_0.5_)O_3_ has been determined to be orthorhombic (*P*bnm) structure with the *a* ^−^ *a* ^−^ *c* ^+^ tilting system, which induces the mixed ½{*ooo*} + ½{*ooe*} + ½{*oee*} superlattice reflections.^[^ ^26^ ^]^ In this work, the X‐ray diffraction patterns (XRD, Figure 2c) clearly show the presence of several superstructure peaks ½(310)_pc_, ½(311)_pc_, ½(320)_pc_, and ½(321)_pc_ at *x* = 0.22. It is believed that the increase of *x* can induce the orthorhombic *P*bnm phase and the oxygen octahedral tilting distortion, as evidenced by the appearance of ½{*oee*}‐type reflection and the increased intensity of superstructure peaks. The apparent (200) peak asymmetry at *x* = 0.22 is also indicative of the existence orthorhombic phase (Figure 2d).

Rietveld refinement was performed on XRD patterns with potential space groups, and two‐phase model “*P*4bm+*R*3c” is used for *x* = 0.00 ceramic, while *x* = 0.10‐0.22 compositions can be identified as three‐phase model “*P*4bm+*P*bnm+*R*3c” (Figure S2 and Table S1, Supporting Information). The *x* = 0.22 ceramic shows the coexistence of 73.2 wt.% *P*bnm, 23.6 wt.% *P*4bm, and 3.2 wt.% *R*3c phases (Figure 2e). Moreover, the average tilting degree of oxygen octahedral can be estimated by *ω* = (180°‐∠B‐O‐B)/2, and *ω*_1_ and *ω*_2_ were used to represent the oxygen octahedral tilting degree in the *P*bnm phase. Large *ω* values over 11.8° for the *P*bnm can be found in the studied ceramics, which is much larger than that of *P*4bm and *R*3c phases in BNT matrix (Figure 2f; Figure S3, Supporting Information). The Raman spectra of BNBCTFT*_x_* ceramics (Figure 2g) display three main regions, which stem from the vibrations of A‐site cations, B─O bonds, and BO_6_ octahedra, respectively. The corresponding peak fitting results reveal a triple splitting feature for both the B‐O and BO_6_ vibration modes, confirming the multiphase structure.^[^ ^27^, ^28^ ^]^ Notably, the B_2_ mode ≈270 cm^−1^ sensitive to O‐B‐O bending motion only and C_2_ mode ≈550 cm^−1^ sensitive to BO_6_ vibration both show a steady softening with the increase of *x* (Figure S4, Supporting Information), implying the bond weakening due to the Fe^3+^ and Ta^5+^ doping. Intriguingly, the broadening of Raman peaks in the wavenumber region of 150‐400 cm^−1^ with the increase of *x* signifies a higher B─O bond disorder in the lattice, while the Raman peaks in 400‐700 cm^−1^ become more intensive, which is usually connected with the increased BO_6_ ordering.

In order to clarify the roles of Fe^3+^/Ta^5+^/Ca^2+^ doping in modulating the crystal structures, DFT calculations are performed (Figure 2h; Figure S5, Supporting Information) and the 2×2×4 BNBT supercells with *R*3c symmetry are constructed as the matrix. The results reveal the effects of Fe^3+^/Ta^5+^/Ca^2+^ doping on suppressing B‐site cation displacement and enhancing random fields, which are responsible for the weakened ferroelectric distortion and ordering. In terms of the oxygen octahedral tilting, it shows that the single A‐site Ca^2+^ doping effectively increases the oxygen octahedral tilting degrees surrounding the doping site while the single B‐site Fe^3+^/Ta^5+^ doping doesn't make a big difference. It is believed that BO_6_ tilting enhancement and ordering are induced to accommodate the continuously decreasing tolerance factor *t* and can mainly be attributed to the introduction of small‐sized Ca^2+^ ions.

### 2.2 Long‐Range‐Ordered Ferroelastic Microdomains and Weakly‐Coupled PNRs

**Figure 3:**
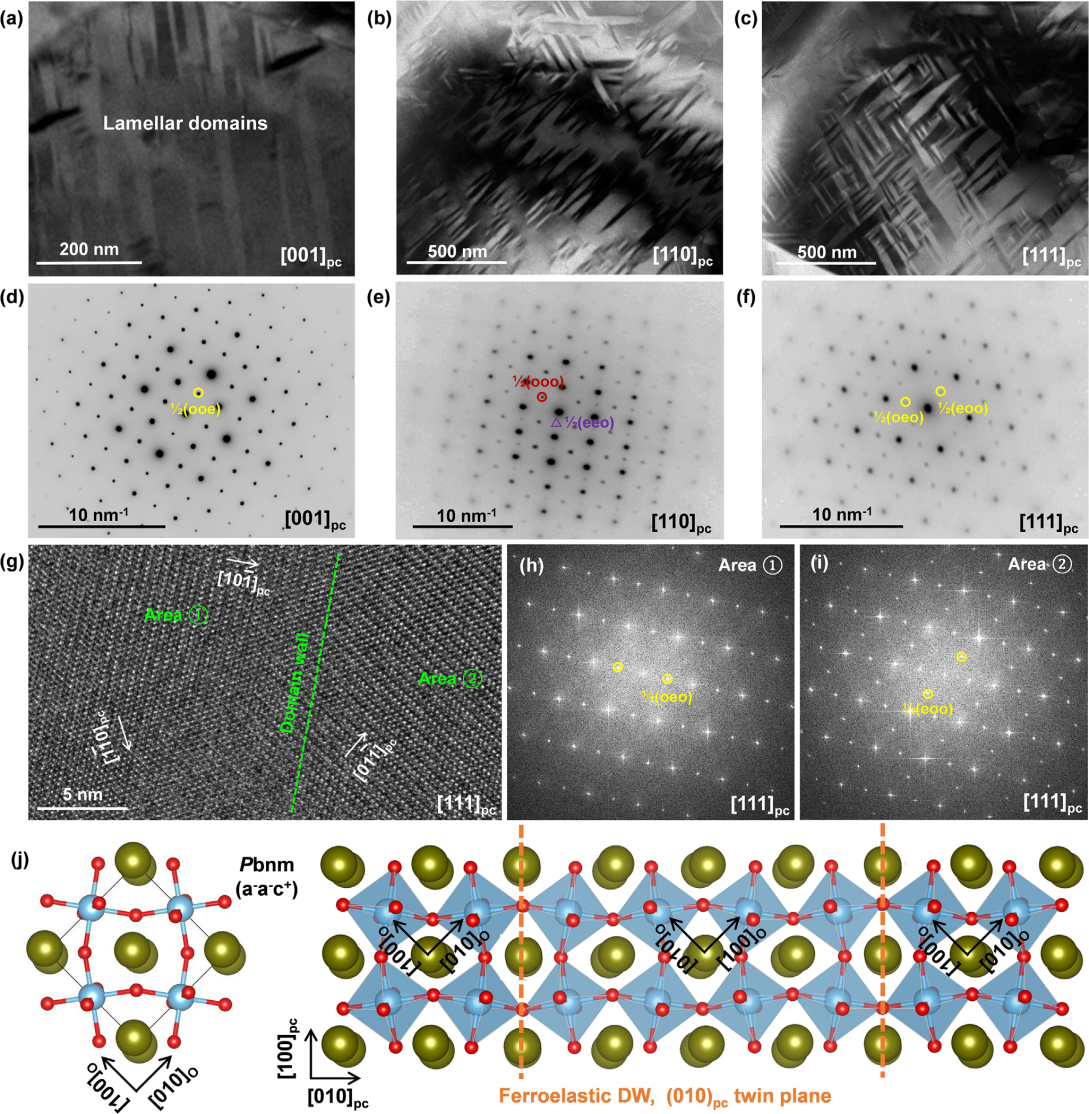
Domain structures and SAED patterns for the *x* = 0.22 ceramic. Low‐magnification TEM images showing the high‐density lamellar domains captured along the a) [001]_pc_, b) [110]_pc_, and c) [111]_pc_ directions. SAED patterns captured along the d) [001]_pc_, e) [110]_pc_, and f) [111]_pc_ directions. g) [111]_pc_‐viewed high‐resolution atomic image and SAED patterns for h) domain area ① and i) ②. j) Atomic configuration of the *P*bnm structure viewed along the [001]_pc_ direction and structure model consisting of two parallel (010)_pc_‐type ferroelastic domain boundary.

To gain an in‐depth understanding for this special high‐entropy relaxor ferroelectric with large antiferrodistortion, the nanostructure details of the *x* = 0.22 ceramic are further characterized by the transmission electron microscopy (TEM). Intriguingly, high‐density lamellar domains with the size of hundreds of nanometers are found to occupy the grains of the *x* = 0.22 ceramic (**Figure** **3**a–c; Figure S6, Supporting Information) instead of the blotchy PNRs found in the PFM analysis (Figure S7, Supporting Information). To explain this paradox, the [001]_pc_, [110]_pc_, and [111]_pc_‐viewed selected‐area‐electron‐diffraction (SAED) patterns for these lamellar domains are characterized (Figure 3d–f). In the [001]_pc_ and [110]_pc_‐viewed SAED patterns, we can observe the existence of strong ½{ooe}, ½{ooo}, and weak ½(eeo) superlattice spots, characteristic of the orthorhombic *P*bnm (*a* ^−^ *a* ^−^ *c* ^+^) phase. More intriguingly, only ½{oeo} and ½{eoo} superlattice spots can be detected in the [111]_pc_‐viewed SAED pattern while ½{ooe} spots are absent, which reveals the ordering of oxygen octahedral tilting because their random distribution would give rise to superlattice spots in all equivalent positions. Furthermore, we carefully analyze the [111]_pc_‐viewed TEM atomic image of the area across the domain boundary. Differential arrangements of double‐period lattice fringes are observed on adjacent sides of the domain boundary, which is along the [10—1]_pc_ direction for area ① and the [01—1] direction for area ② (Figure 3g). The corresponding SAED pattern for area ① only displays ½(oeo) superlattice spots (Figure 3h), and in comparison, the ½(eoo) superlattice spots are evident for area ② (Figure 3i). These results directly indicate the oxygen octahedral tilting ordering in the *x* = 0.22 ceramic and the resulting visible lamellar ferroelastic domains.

**Figure 4:**
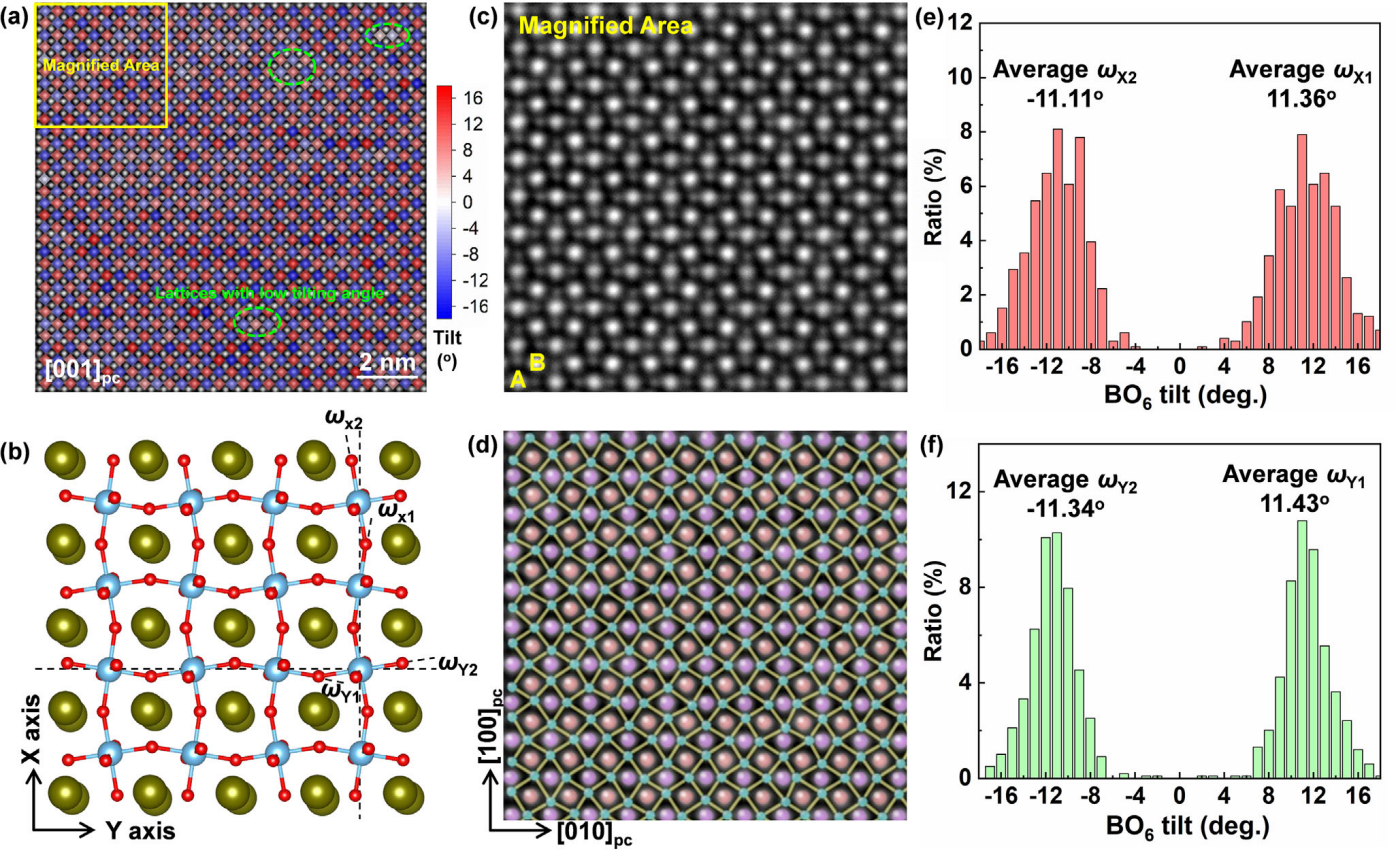
Oxygen octahedral tilting in the *x* = 0.22 ceramic. a) [001]_pc_‐viewed iDPC‐STEM image showing the in‐phase oxygen octahedral tilting ordering. b) Structure model of *P*bnm phase showing the rippling of O‐sublattice. c) Magnified view showing the A, B, and O columns and d) the O‐sublattice network. e,f) Octahedral tilting angle distribution along X and Y directions.

In the typical *P*bnm perovskite system CaTiO_3_, similar ferroelastic domains have been reported and the emergence of domain boundary can be attributed to the reconfiguration of oxygen octahedral tilting.^[^ ^29^ ^]^ When imaged along the [001]_pc_ direction, the (010)_pc_‐type ferroelastic domain boundary is existed as a mirror plane (Figure 3j). High‐resolution scanning TEM with high‐angle annular dark‐field imaging (HAADF‐STEM) and integrated differential phase contrast imaging (iDPC‐STEM) were further performed to visualize the A, B, and O columns in the *x* = 0.22 ceramic. The [001]_pc_‐viewed iDPC‐STEM image exhibits a long‐range ordering characteristic of in‐phase oxygen octahedral tilting with periodically alternating anticlockwise and clockwise tilting (**Figure** **4**a) and the tilting angle is evaluated by measuring the rippling of O‐sublattice along both X and Y directions (Figure 4b). The magnified view clearly shows the positions of the A‐site and B‐site atoms (Figure 4c), as well as the O‐sublattice network (Figure 4d). The tilting angle along X or Y direction shows a distinct bilateral distribution with the average absolute value larger than 11° (Figure 4e,f). In the (001)_pc_ plane, such a periodically alternating in‐phase oxygen octahedral tilting with high tilting angle should be attributed to the *P*bnm (*a* ^−^ *a* ^−^ *c* ^+^) phase, and it is the origin of ferroelastic microdomain walls to minimize elastic strain energy. Furthermore, it should be noted that within this region, the tilting angles of the oxygen octahedra are not constant but exhibit an approximately normal‐like distribution, with a minority of lattices showing low tilting. This phenomenon is commonly observed in the lamellar ferroelastic domains (Figure S8, Supporting Information), and should be attributed to the atomic‐scale chemical heterogeneity induced by the high‐entropy effect. The lattices with low tilting arise from accommodating the competing bonding requirements, which could correspond to the coexistent *P*4bm and *R*3c phases. On the other hand, the ferroelastic domain boundary is hard to be distinguished from oxygen octahedral tilting information in the [001]_pc_‐viewed iDPC‐STEM image.

**Figure 5:**
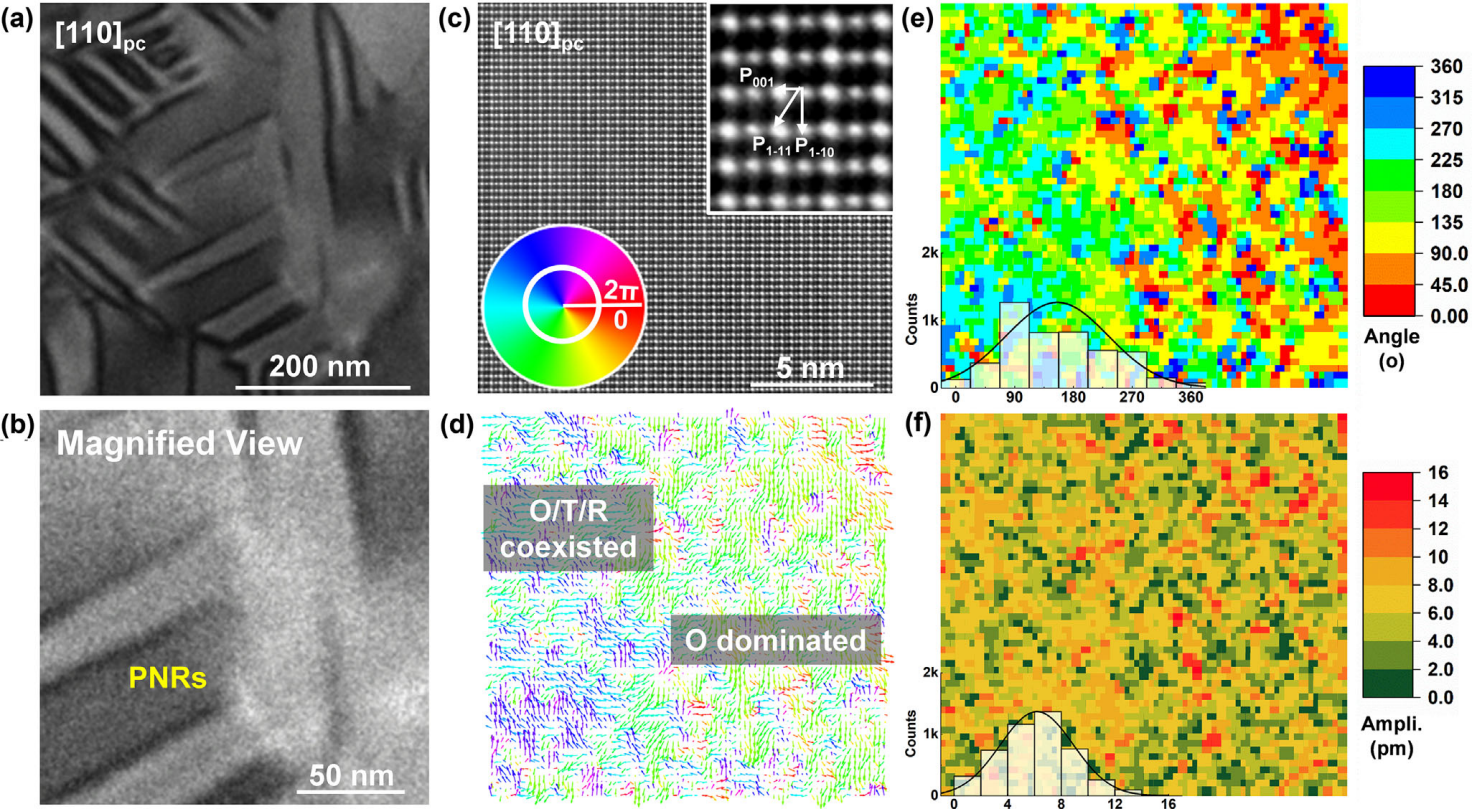
Polar nanoregions in the *x* = 0.22 ceramic. a,b) Low‐magnification bright‐field TEM images reveal a blotchy domain morphology. c,d) [110]_pc_‐viewed HAADF‐STEM atomic image showing the polarization vector distribution. Inset shows the polarization vector directions along [001]_pc_, [1—10]_pc_, and [1—11]_pc_. Statistic distribution of e) angle and f) magnitude of the polarization vectors.

The ideal *P*bnm phase is paraelectric, characterized by collective antipolar displacements of A‐site cations along the along [110]_PC_/[1—10]_PC_ directions.^[^ ^30^ ^]^ However, temporal fluctuations in structural distortions induced by the high‐entropy effect, coupled with local strain and electric fields arising from compositional disorder, could locally break spatial inversion symmetry. Furthermore, alongside the orthorhombic *P*bnm phase, the *x* = 0.22 ceramic also contains polar tetragonal *P*4bm and rhombohedral *R*3c phases. Thus, beyond analyzing oxygen octahedral distortions, it is also critical to examine the distribution of cation displacements or polarization vectors within the ceramic.^[^ ^31^ ^]^ The polarization vector distribution can be obtained by calculating the displacement of the B(A) site cations relative to the center of four corners of A(B) site cations using customized MATLAB scripts. The polarization vector map corresponding to Figure 4a viewed along the [001]_pc_ direction (Figure S9, Supporting Information) shows the randomly distributed PNRs, in which the [100]_pc_ and [110]_pc_‐oriented polarization vectors can be recognized, corresponding to the tetragonal and orthorhombic/rhombohedral phases, respectively. Furthermore, [110]_pc_‐viewed TEM and HAADF‐STEM characterizations were performed to identify the cation displacement along the [001]_pc_ (tetragonal *P*4bm phase), [1—10]_pc_ (orthorhombic *P*bnm phase), and [1—11]_pc_ (rhombohedral *R*3c phase) directions. The low‐magnification bright‐field TEM images reveal a blotchy domain morphology within the lamellar ferroelastic domains (**Figure** **5**a,b), corresponding to small polar nanoregions (PNRs), consistent with the PFM results. It implies that long‐range ordered oxygen octahedral tilting within the lamellar domains coexisted with the substructure of randomly embedded PNRs. Within a typical stripe domain, the PNRs showing the [001]_pc_‐, [1—10]_pc_‐, and [1—11]_pc_‐oriented cation displacements can be clearly recognized, which display the sizes of 1‐3 nm, as shown in the [110]_pc_‐viewed HAADF‐STEM image (Figure 5c,d). It indicates the effective disruption of ferroelectric ordering due to the incorporation of Ca^2+^, Fe^3+^, and Ta^5+^. Besides, there are regions dominated by the orthorhombic phase alongside regions exhibiting distinct coexistence of the orthorhombic/tetragonal/rhombohedral phases, which should be attributed to the local composition fluctuations, as indicated by the energy dispersive spectroscopy (EDS) analysis for elemental mapping in the atomic scale (Figure S10, Supporting Information). On the whole, such a polar state with ultrasmall PNRs and strong direction/length fluctuations in polarization vectors (Figure 5e,f) is highly desired to reduce the polarization anisotropy and field‐induced polarization hysteresis by a fast polarization reorientation.

### 2.3 Excellent Dielectric Energy Storage Performance

**Figure 6:**
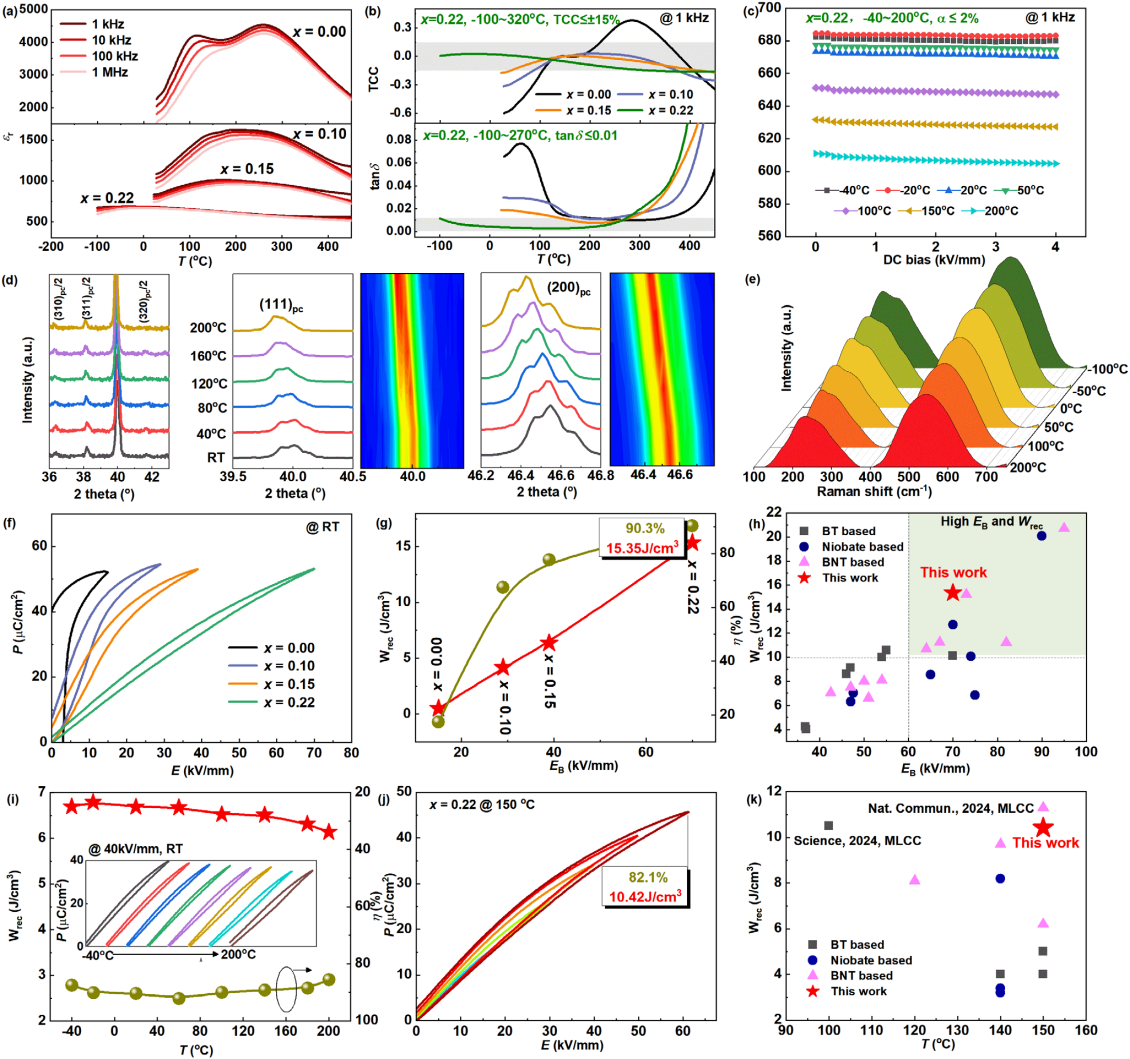
Temperature‐dependent dielectric, polarization, and energy storage performance of the BNBCTFT*_x_* ceramics. a) Temperature‐dependent dielectric spectra measured at different frequencies. b) TCC‐*T* curves and tan*δ*‐*T* curves of the BNBCTFT*_x_* ceramics measured at 1 kHz. c) DC bias‐dependent *ε*_r_ measured at different temperatures. d) Temperature‐dependent XRD patterns and e) Raman spectra. f) Room‐temperature *P*‐*E* loops, g) *W*_rec_ and *E*_B_ for all samples. h) Comparison of room‐temperature *W*_rec_ and *E*_B_ between the *x* = 0.22 ceramic and other lead‐free ceramics. i) Temperature‐dependent *P*‐*E* loops, *W*_rec_, and *η* measured at 40 kV mm^−1^. j) Electric‐field‐dependent *P*‐*E* loops measured at 150 °C. k) Comparison of high‐temperature *W*_rec_ between the *x* = 0.22 ceramic and other lead‐free ceramics.

The temperature‐dependent dielectric spectra of BNBCTFT*_x_* ceramics (**Figure** **6**a) exhibit obvious dielectric relaxation and the increase of *x* leads to the broadening and flattening of dielectric peaks. Based on the 1/*ε*_r_‐*T* plots, nonlinear Vogel‐Fulcher fitting of dielectric spectra, and analysis by the modified Curie‐Weiss law (Figure S11, Supporting Information), the increase of *x* can cause a significant decrease in the characteristic temperatures *T*_B_, *T*_m_, and *T*_f_, as well as an increase in the degree of diffuseness *γ*. The above phenomena signal the disruption of long‐range ferroelectric ordering and the weakening of dipole‐dipole interaction with the increase of *x*. Specially, when *x* = 0.22, the *T*_m_ drops below room temperature (−4 °C) and the *T*_f_ is as low as −58 °C, revealing a room‐temperature SPE state for this composition. The thermal stability of *ε*_r_ can be evaluated by the temperature coefficient of capacitance (TCC):

(1) $$\mathrm{TCC}=\frac{\Delta \varepsilon_{r}}{\varepsilon_{r\;\left(\mathrm{base}\right)}}=\frac{\varepsilon_{r\;\left(T\right)}-\varepsilon_{r\;\left(\mathrm{base}\right)}}{\varepsilon_{r\;\left(\mathrm{base}\right)}}$$

The increase of *x* greatly enhances the thermal stability of *ε*_r_, quantitatively, the *x* = 0.22 ceramic exhibits a relatively high *ε*_r_ (25 °C, 1 kHz) of 676 with the TCC ≤ ±15% over a wide temperature range of –100–320 °C. Meanwhile, the dielectric loss tan*δ* at 1 kHz can be maintained less than 0.02 over –100–300 °C (≤0.01 over –100–270 °C) with the tan*δ* (25 °C) of 0.0037 (Figure 6b). Comparison of *ε*_r_, tan*δ*, and their temperature stability between the *x* = 0.22 ceramic and reported BaTiO_3_‐based, niobate‐based, and BNT‐based ceramics demonstrates the advantages of the *x* = 0.22 ceramic in achieving the relatively high *ε*_r_ and low tan*δ* simultaneously over a wide temperature range (Figure S12, Supporting Information). We also analyzed the DC‐bias stability (Figure 6c) of dielectric properties for the *x* = 0.22 ceramic at different temperatures, where the coefficient *α* is used:

(2) $$\alpha =\frac{\varepsilon_{r}\left(0\right)-\varepsilon_{r}\left(E\right)}{\varepsilon_{r}\left(0\right)}$$

For commercial ceramic capacitors, the value of *α* could reach 20–50% under a DC bias field of 4 kV mm^−1^ due to the field‐induced domain switching, which however, is only ≈2% for our *x* = 0.22 ceramic over the wide temperature range of ‐40–200 °C and the tan*δ* is below 0.01 at the same time (Figure S13, Supporting Information)

Temperature‐dependent XRD (Figure 6d) and Raman analyses (Figure 6e) were measured to probe the evolution of the crystal phase structure and lattice symmetry with temperature in the *x* = 0.22 ceramic. From the XRD results, the ½{310}_pc_, ½{311}_pc_, and ½{320}_pc_ superlattice diffraction peaks persist within room temperature‐200 °C and their intensities almost unchanged with increasing temperature. Moreover, the {111} _pc_ and {200} _pc_ peak splitting slightly diminishes with increasing temperature and all diffraction peaks shift toward lower angles, indicating a lattice expansion and a trend toward higher symmetry. Rietveld refinement using the three‐phase model “*P*bnm+*P*4bm+*R*3c” is performed on the XRD data collected at 200 °C (Figure S14, Supporting Information). The ceramic shows the coexistence of 66.3 wt.% *P*bnm, 31.2 wt.% *P*4bm, and 2.5 wt.% *R*3c phases at 200 °C. Increasing temperature to 200 °C only leads to a slight increase of the *P*4bm phase. Furthermore, Raman spectra acquired across ‐100–200 °C exhibit similar spectral profiles (Figure S15, Supporting Information). Significantly, both the B─O bond stretching vibrations and the BO_6_ octahedral vibrational modes display a triplet splitting feature. This characteristic splitting provides corroborating evidence for the similar multiphase coexistence structure within ‐100–200 °C. Based on these results, it can be concluded that the stable crystal structure of the *x* = 0.22 ceramic, especially the temperature‐insensitive *P*bnm phase, plays an important role in the stable dielectric performance over a wide temperature range. The long‐range ferroelastic distortion in the *x* = 0.22 ceramic, established by cooperative oxygen octahedral tilting, creates a structurally rigid and thermally stable backbone. This framework generates a pervasive, anisotropic strain field that fundamentally alters the energy landscape for PNRs. It imposes a restoring force, suppressing their coalescence and growth upon cooling by elevating the energy barrier for domain coarsening. Consequently, the PNRs remain as slowly‐freezing, weakly‐coupled entities with a low freezing temperature (*T*_f_≈–58 °C), which is the core of enhanced relaxor characteristics. This strain‐field‐mediated pinning effect, synergizing with the local random fields from the high‐entropy composition, effectively decouples the polarization dynamics from thermal agitation. This mechanism promotes a stable dielectric response over a broad temperature range.

On the other hand, the well‐saturated unipolar *P*‐*E* loop for *x* = 0.00 ceramic is transformed to slim *P*‐*E* loops with the increase of *x* (Figure 6f, measured at room temperature and 10 Hz), related to the enhanced domain activity and polarization ergodicity. Besides, obvious polarization saturation delay is achieved, that is, higher electric fields are required to induce the long‐range ferroelectric state. As a result, a high *P*_m_ of 53.12 µC cm^−2^ and a low *P*_r_ of 1.73 µC cm^−2^ are achieved in the *x* = 0.22 ceramic at the large *E*_B_ of 70 kV mm^−1^, leading to an excellent energy storage performance with *W*_rec_ of 15.35 J cm^−3^ and *η* of 90.3 % (Figure 6g), which exhibits a greater advantage among the lead‐free dielectric energy storage ceramics, including BaTiO_3_‐,^[^ ^4^, ^5^, ^9^, ^11^, ^32^, ^33^, ^34^ ^]^ niobate‐,^[^ ^12^, ^21^, ^35^, ^36^, ^37^, ^38^, ^39^, ^40^ ^]^ and BNT‐based^[^ ^6^, ^10^, ^13^, ^20^, ^41^, ^42^, ^43^, ^44^, ^45^, ^46^, ^47^ ^]^ systems (Figure 6h). High *E*_B_ is indispensable to excite a high *P*_m_ and the resulting high *W*_rec_ in the *x* = 0.22 ceramic because i) the incorporation of Ca^2+^, Fe^3+^, and Ta^5+^ leads to weakened spontaneous polarization, ii) high ferroelastic distortion and lattice compression suppresses the B‐site cation movement under electric fields, iii) SPE state links with weakened PNRs interaction and strong random fields. The average grain size of BNBCTFT*_x_* ceramics increases with the increase of *x* (Figure S16, Supporting Information), thus grain refinement to enhance *E*_B_ is not inapplicable in our work. Here, three factors are responsible for the substantial increase in *E*_B_. First, the polymorphic PNRs in the high‐entropy composition with *x* = 0.22 possess a slim hysteresis loop, significantly reducing polarization hysteresis loss under high electric fields, thus restricting the heat dissipation and thermal breakdown probability. Second, this specific composition is the key to achieving a lattice with large ferroelastic distortion and a SPE‐like state under strong random fields. This unique state leads to an ultra‐low electrostrictive strain (reduced from ≈0.356% to ≈0.012% at 10 kV mm^−1^, Figure S17, Supporting Information), which effectively minimizes intergranular deformation and is conductive to the electromechanical breakdown strength. Moreover, the *in‐situ* temperature dependent DC resistivity (*ρ*_DC_) measurement shows that the increase of *x* can effectively increase the *ρ*_DC_ (Figure S18, Supporting Information). Below 125 °C, the *ρ*_DC_ of the *x* = 0.22 ceramic can reach as high as 10^13^Ω•cm, beneficial for the electrical breakdown strength. The pronounced chemical disorder arising from the multi‐cation complexity acts as strong scattering centers for charge carriers (electrons/holes). It increases the probability of electron‐lattice collisions, effectively impeding their long‐range migration.^[^ ^4^ ^]^ The X‐ray photoelectron spectroscopy (XPS) analysis (Figures S19 and S20, Supporting Information) explains the reasons for the decreased *ρ*_DC_ with *x* increasing when the temperature is higher than 125 °C, which arise from the thermally‐activated oxygen vacancies.

In terms of the frequency stability of dielectric energy storage properties (Figure S21, Supporting Information), it is observed that the *ε*_r_ exhibits the variation rate of less than 6 % and the tan*δ* is lower than 0.02 over 100 Hz–1 MHz when temperatures reside within ‐40–200 °C, attributed to the strong ergodicity of the *x* = 0.22 ceramic with the presence of highly‐dynamic PNRs in the temperature range. At room temperature, the frequency stability of energy storage performance over 1–200 Hz is also satisfactory, exhibiting the *W*_rec_ of 6.42–6.72 J cm^−3^ and *η* of 89.7–93.2%, respectively. Moreover, it was found that the *P*‐*E* loops (measured at 10 Hz) all exhibit a slim shape in the tested temperature range of ‐40–200 °C, and the calculated *W*_rec_ and *η* vary in the range of 6.50±0.30J cm^−3^ and 90±3.5%, respectively, demonstrating the excellent thermal stability (Figure 6i). It was also observed that the *η* decreased at both low (e.g., −40 °C) and high (e.g., 200 °C) temperatures compared to its room‐temperature value. The reduction at low temperature is primarily attributed to the increased size and enhanced interaction of the PNRs in the *x* = 0.22 ceramic. In contrast, the efficiency decline at elevated temperatures is mainly due to the degradation of insulation resistivity. To examine the high‐temperature energy‐storage performance, the electric‐field‐dependent *P*‐*E* loops of the *x* = 0.22 ceramic were measured at 150 °C (Figure 6j) and a high *W*_rec_ of 10.42J cm^−3^ with *η* of 82.1% was achieved at the large *E*_B_ of 61 kV mm^−1^, superior to most lead‐free dielectric energy storage ceramics^[^ ^4^, ^5^, ^10^, ^11^, ^12^, ^16^, ^22^, ^39^, ^42^, ^47^ ^]^ (Figure 6k). The stable polarization (dielectric) response (*ε*_r_≈630 and tan*δ*≈0.0026 at 150 °C and 1 kHz) and high‐enough insulation resistivity at elevated temperatures (>10^12^Ω•cm at 150 °C) are responsible for the exceptional high‐temperature energy storage performance.

In summary, we constructed a high‐entropy ferroelastic‐ferroelectric hybrid perovskite system Bi_0.3666_Na_0.3666_Ba_0.0468_Ca_0.22_Ti_0.78_Fe_0.11_Ta_0.11_O_3_, for ultrahigh and temperature‐insensitive dielectric energy storage. Building upon the high‐entropy effect, which induces atomic‐scale polar heterogeneity of dipoles, we employed precise compositional control to promote cooperative oxygen octahedral tilting. This strategy yielded a distinctive hybrid microstructure: ferroelastic microdomains spanning hundreds of nanometers, embedded with a substructure of randomly dispersed PNRs measuring 1–3 nm in size. Herein, reducing the tolerance factor *t* and incorporating the key element Ca were crucial for inducing the substantial oxygen octahedral tilting distortion and achieving long‐range order, an approach potentially transferable to other perovskite systems. This unique hybrid architecture endows the ceramic with superior dielectric energy storage performance and exceptional temperature stability. The performance enhancement originates from several key mechanisms: (i) Reduced polarization hysteresis facilitated by atomic‐scale polar heterogeneity; (ii) Delayed polarization saturation arising from large ferroelastic distortion and the SPE state under strong random fields; (iii) Enhanced electrical breakdown strength achieved through minimized polarization loss, reduced conduction loss, and suppressed electrostrictive strain; (iv) Improved structural thermal stability enabled by the additional stress field generated by the long‐range ferroelastic distortion. Consequently, a high *W*_rec_ of 15.35 J cm^−3^ with *η* of 90.3% are achieved at 70 kV mm^−1^. The ceramic also exhibits outstanding temperature‐insensitive energy storage performance (*W*_rec_ > 6 J cm^−3^ and *η* > 85% over ‐40–200 °C) and dielectric properties (TCC ≤ ±15% over –100–320 °C and tan*δ*≤0.01 over –100–270 °C).

## 3 Experimental Section

### Ceramic Preparation

The (Bi_0.47_Na_0.47_Ba_0.06_)_1‐_ *_x_* Ca*_x_* Ti_1‐_ *_x_* Fe_0.5_ *_x_* Ta_0.5_ *_x_* O_3_ (BNBCTFT*_x_*)ceramics were prepared by the solid‐state method starting from high‐purity raw materials (> 99%) of Bi_2_O_3_, Na_2_CO_3_, BaCO_3_, CaCO_3_, TiO_2_, Fe_2_O_3_, and Ta_2_O_5_. The weighed powers were mixed by milling in ethanol for 8 h at 300 rpm, and then the dried mixed powders were calcinated at 900 °C for 3 h. The BNBCTFT*_x_* calcined powders were milled in ethanal for 12 h at 400 rpm, and then mixed with 3 wt.% PVB. Afterwards, pellets with a diameter of 10 mm were pressed and sintered at 1180 °C for 2 h to gain the BNBCTFT*_x_* ceramics.

### Structure Characterization

The phase structures were tested by a D8 Advance X‐ray diffractometer (XRD, Bruker AXS, Germany, Cu‐Kα radiation) equipped with a heating stage. Raman spectra were collected by a Horiba LabRAM HR Evolution confocal Raman microscope (HORIBA Scientific, France) equipped with a heating stage. The ceramics grain analysis was performed on a scanning electron microscope (SEM, MIRA3, TESCAN, Czech Republic). The TEM specimen was prepared using focused ion beam (FIB) milling. Domain morphology, SAED patterns, HAADF‐STEM and iDPC‐STEM images were acquired using a double spherical aberration‐corrected transmission electron microscope (Thermo Fisher Spectra 300, USA). Elemental distribution mapping was performed using the integrated EDS system. Based on the acquired STEM images, the polarization vector, polarization magnitude, polarization angle, and oxygen octahedral tilting were calculated and extracted using custom MATLAB scripts. PFM measurement was performed on atomic force microscope (AFM, NanoManTM VS, Veeco, USA). Before the PFM measurements, the ceramics were thoroughly polished and thinned to ≈0.10 mm thickness.

### Electrical Characterization

Ceramic pellets, polished to a thickness of 0.3 mm, were coated with silver paste electrodes for dielectric and *S*‐*E* characterization. Temperature‐ and frequency‐dependent dielectric spectra and complex impedance plots were acquired using a commercial dielectric measurement system (DMS‐500, Partulab Technology Co., Ltd, China). The DC‐bias stability of dielectric response was characterized by a dielectric tunability test system (DSP0200, Tongguo Technology, China). Temperature‐dependent DC resistivity was measured using a resistivity measurement system (RMS‐1000, Partulab Technology Co., Ltd, China). For *P*‐*E* and *I*‐*E* characterization, separate pellets were polished to a thickness of 0.08 mm and electrodes with a diameter of 1.0 mm were deposited by sputtering gold (Au). *S*‐*E*, *P*‐*E* and *I*‐*E* curves were characterized using a ferroelectric tester (TF Analyzer 2000, AixACCT Systems GmbH, Germany).

### First‐Principles Calculations

First‐principles calculations were performed by the density functional theory (DFT) using the Vienna Ab‐initio Simulation Package (VASP) package. The generalized gradient approximation (GGA) with the Perdew‐Burke‐Ernzerhof (PBE) functional was used to describe the electronic exchange and correlation effects. The simulation was run with a cutoff energy of 500 eV throughout the computations. The energy criterion was set to 10^−5 ^eV in iterative solution of the Kohn‐Sham equation. The Brillouin zone integration was performed using a 4×4×4 k‐mesh. All the structures were relaxed until the residual forces on the atoms have declined to less than 0.02 eV/Å. The simulation was carried out within the 2×2×2 supercell (containing 40 atoms) of the Bi_4_Na_3_Sr_1_Ti_8_O_24_, Bi_4_Na_3_La_1_Ti_8_O_24_, and Bi_4_Na_3_Ca_1_Ti_8_O_24_ lattice with *R*3c symmetry, and the 2×2×4 supercell (containing 80 atoms) of the Bi_7_Na_8_Ba_1_Ti_16_O_48_, Bi_7_Na_8_Ba_1_Ti_15_Fe_1_O_48_, and Bi_7_Na_8_Ba_1_Ti_15_Ta_1_O_48_, and Bi_7_Na_7_Ca_1_Ba_1_Ti_16_O_48_ lattice with *R*3c symmetry, respectively.

## Conflict of Interest

The authors declare no conflict of interest.

## Author Contributions

The work was conceived and designed by X.F.Z. X.F.Z. Y.C.S., and Z.M.H. fabricated the samples, characterized the microstructures, tested the electrical properties, and processed related data assisted by Y.Z. and H.L. The TEM and HAADF‐STEM images were processed by H.Q. The first‐principles calculations were processed by X.F.Z. The paper was drafted by X.F.Z., and revised by H.Q. and D.Z. All authors participated in the data analysis and discussions.

## Supporting information



Supporting Information

## Data Availability

The data that support the findings of this study are available from the corresponding author upon reasonable request.
