# Supplementary material for: High‐Entropy Ferroelectric‐Ferroelastic Hybrid for Ultrahigh and Temperature‐Insensitive Dielectric Energy Storage
Source: Adv Sci (Weinh). 2025 Nov 17;13(6):e18725. doi: 10.1002/advs.202518725 (PMC12866784; doi:10.1002/advs.202518725)
Supplement: Supplementary file 1 — Supporting Information [file ADVS-13-e18725-s001.docx]

**Supporting Information for**

**High-Entropy Ferroelectric-Ferroelastic Hybrid for Ultrahigh and Temperature-Insensitive Dielectric Energy Storage**

Xuefan Zhou ^a^, He Qi ^b,^ *, Yingchun Su ^a^, Zhimin Huang ^a^, Yan Zhang ^a^, Hang Luo ^a^, Dou Zhang ^a,^ *

^a^ Powder Metallurgy Research Institute, State Key Laboratory of Powder Metallurgy, Central South University, Changsha, Hunan 410083, China.

^b^ School of Materials Science and Technology, Hainan University, Hainan 570228, China.

*Corresponding author

E-mail address:

qihe@hainanu.edu.cn (H. Q.), [dzhang@csu.edu.cn](mailto:dzhang@csu.edu.cn) (D.Z.)

**Supplementary Fig. 1 Configurational entropy (*S*_config_) of the BNBCTFT*_x_* systems.**

**

**

The configuration entropy (*S*_config_) of the BNBCTFT*_x_* ceramics was calculated based on the equation:

$$S_{\mathrm{config}}=-R\left[ \left( \sum_{i=1}^{N} x_{i}\ln x_{i} \right)_{cation-site}+\left( \sum_{j=1}^{M} x_{j}\ln x_{j} \right)_{anion-site} \right]$$

where R is the ideal gas constant (R = 8.314 J K^-1^ mol^-1^), N (M) means the number of atomic species, and $x_{i}$ ($x_{j}$) is the atomic percentage. An *S*_config_ of more than 1.5*R* is generally defined as high-entropy^[[1](#_ENREF_1)]^, therefore, high-entropy system was achieved with *x* ≥ 0.10.

**Supplementary Fig. 2 Rietveld refinement of room-temperature XRD patterns for the BNBCTFT*_x_* ceramics.** **a,** *x* = 0. **b,** *x* = 0.10. **c,** *x* = 0.15. **d,** *x* = 0.22.


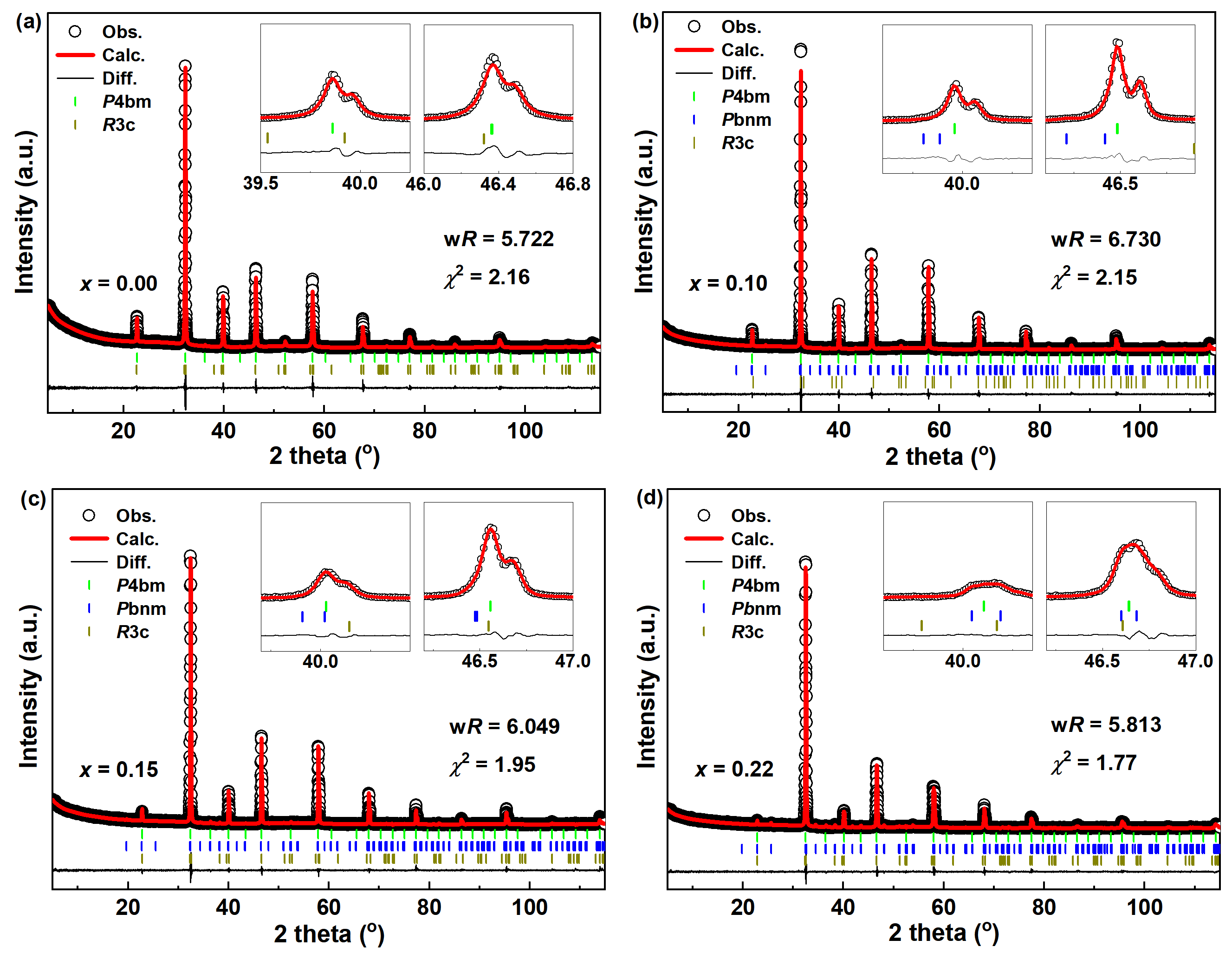


In order to quantify the phases, Rietveld refinement was conducted by the GSAS software. The phase model used gives rise to reliable refinement results, as reflected in the convincible R-factors and goodness-of-fit index χ^2^. The results indicated that the *x* = 0.00 ceramic is dominated by *P*4bm phase and the increase of *x* promotes the formation of *P*bnm phase.

**Supplementary Fig. 3 Structure model of *P*4bm, *R*3c, and *P*bnm phase achieved from the** **Rietveld refinement results.**


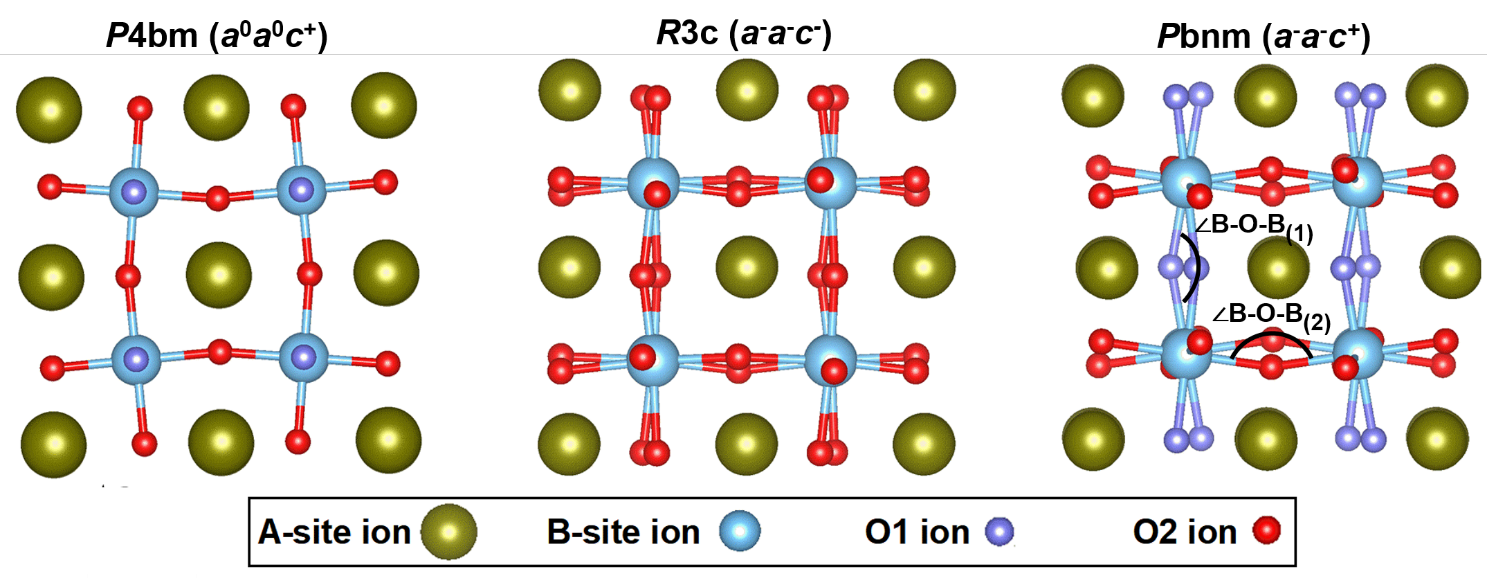


The tilt degree of oxygen octahedron can be estimated by using *ω* = (180^o^-∠B-O-B)/2. The *P*4bm structure shows the *a*^0^*a*^0^*c*^+^ oxygen octahedron tilt system*.* The *R*3c structure shows the *a*^-^*a*^-^*a*^-^ oxygen octahedron tilt system*.* The *P*bnm structure shows the *a*^-^*a*^-^*c*^+^ oxygen octahedron tilt system with high tilt degree of *ω*_1_ and *ω*_2_*.*

**Supplementary Fig. 4 Variations of the Raman mode position with the increase of *x* for the BNBCTFT*_x_* ceramics.**





**Supplementary Fig. 5 DFT calculations of crystal structures for BNBT, Fe-doped BNBT and Ta-doped BNBT with *R*3c symmetry.** **a,** 2×2×4 BNBT, BNBT-Fe and BNBT-Ta supercells with *R*3c symmetry after structural relaxation and the distribution of B-site atom polarization vectors. **b,** B-site atom polarization vector magnitude and **c,** ∠B-O-B magnitude for the BNBT, BNBT-Fe, and BNBT-Ta supercells.


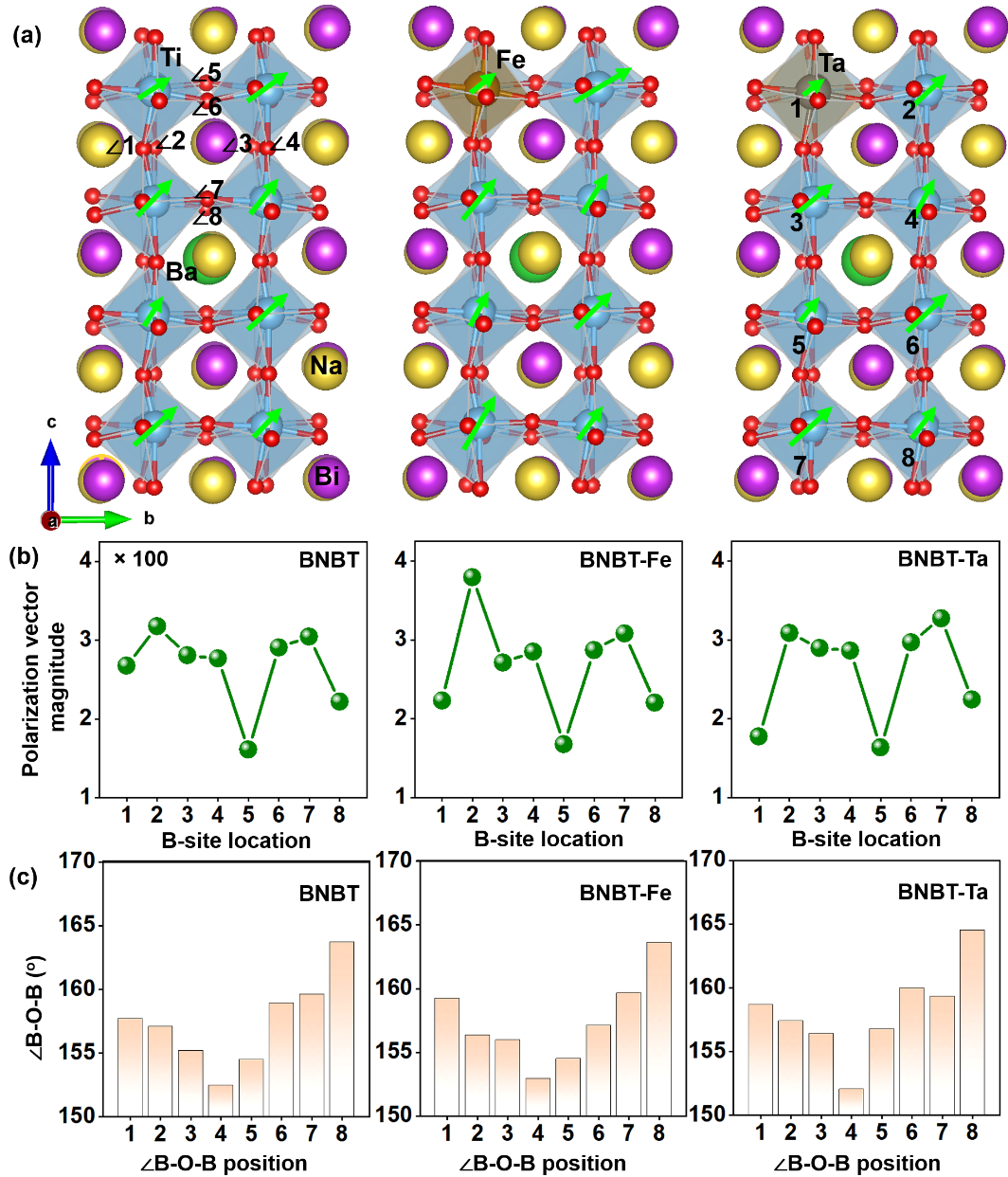


Herein, DFT calculations are performed to clarify the effects of Fe^3+^/Ta^5+^/Ca^2+^ doping on the crystal structure, B-site atom polarization vector magnitude, and ∠B-O-B magnitude. After structural relaxation, the refined atom configurations can be used to calculate the B-site atom polarization vector at different locations. First, the coordinates of each B site atom ($\vec{r}_{B}$) and the surrounding six oxygen atoms ($\vec{r}_{O_{i}}$) are extracted. Then, the geometric center of the BO_6_ octahedron can be calculated as $\frac{1}{6}\sum_{i} \vec{r}_{O_{i}}$. Finally, the B-site atom polar displacement vector ($\vec{D}_{B}$) is obtained by the following equation:

$$\vec{D}_{B}=\vec{r}_{B}-\frac{1}{6}\sum_{i} \vec{r}_{O_{i}}$$

The B-site atom polarization vector length distribution demonstrated that the ferroelectric distortion at the Fe^3+^/Ta^5+^ doping sites decreased significantly. Further, the different ferroelectric distortion between the doping site and surrounding matrix reveals the direction disturbance of ferroelectric ordering. The results show that Fe^3+^/Ta^5+^ doping allows for suppressing the spontaneous polarization and disrupting the ferroelectric ordering. Besides, A-site Ca^2+^ doping can suppress the B-site atom displacement surrounding the doping site and also disturb the polar ordering. By estimating the tilt degree of oxygen octahedron using *ω* = (180^o^-∠B-O-B)/2, it is found that the A-site Ca^2+^ doping plays a vital role in enhancing the BO_6_ tilt in comparison with the B-site Fe^3+^/Ta^5+^ doping.

**Supplementary Fig. 6 Low-magnification TEM images showing the high-density lamellar domains in the *x* = 0.22 ceramic.**

**
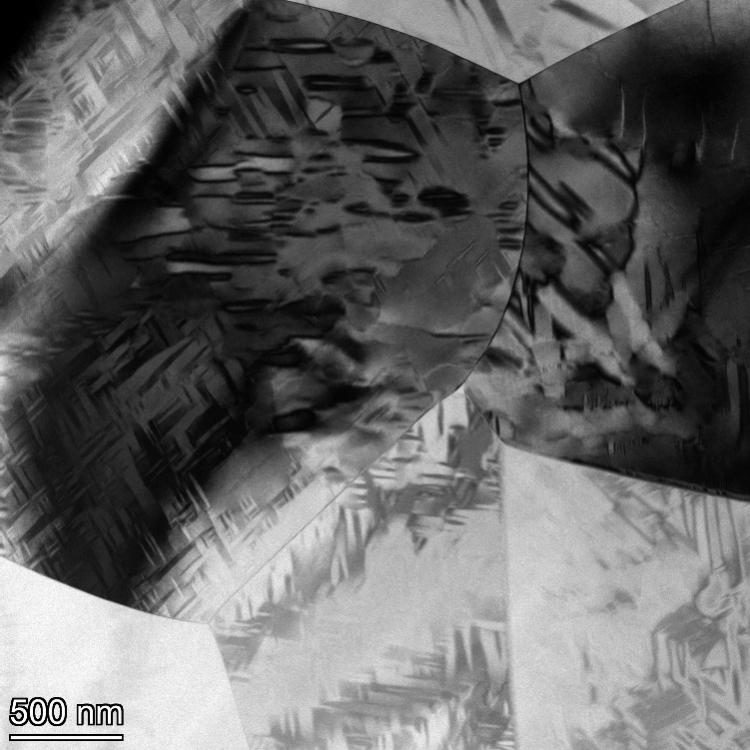
**

**Supplementary Fig. 7 PFM measurement results of the BNBCTFT*_x_* ceramics.** **a,** PFM phase image and **b,** amplitude image for the *x* = 0 ceramic. **c,** PFM phase image and **d,** amplitude image for the *x* = 0.10 ceramic. **e,** PFM phase image and **f,** amplitude image for the *x* = 0.22 ceramic.


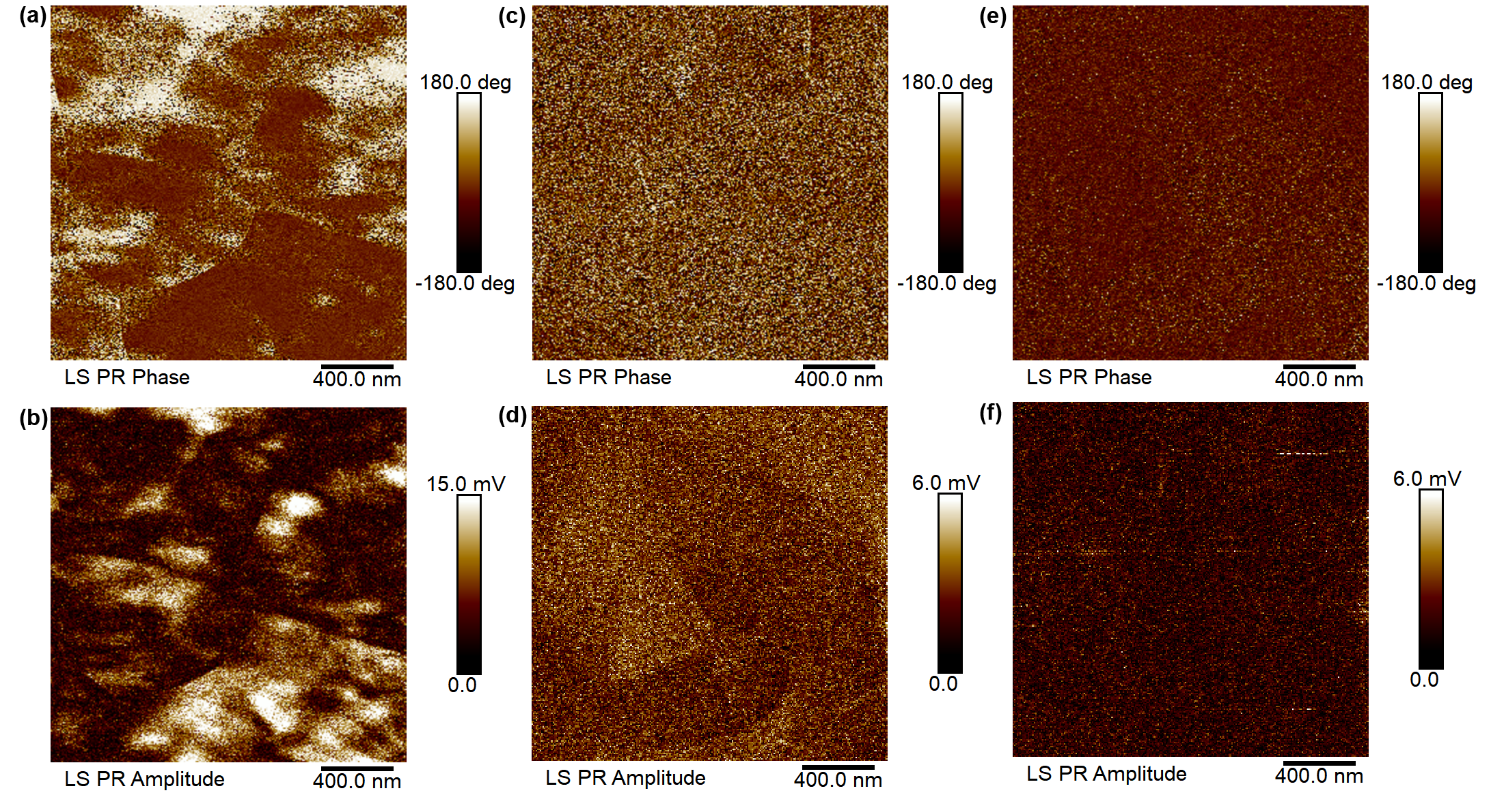


PFM amplitude images exhibit the strength of the piezoelectric response, while the contrast in phase images represents the polarization orientations. It can be seen that the *x*=0.00 ceramic showed larger-sized blocky ferroelectric domains with strong and clear contrasts. With the increase of *x*, the ferroelectric domains became smaller and irregular, accompanied by the appearance of PNRs. The piezoresponse amplitude also gradually decreased. Especially, no clear patterns of domain structures can be observed when *x*=0.22, which may be ascribed to the tiny nanodomains or PNRs for the resolution of PFM.

**Supplementary Fig. 8 Oxygen octahedral tilting ordering in the ferroelastic domains of the *x* = 0.22 ceramic.** **a,** [001]_pc_-viewed iDPC-STEM images showing the A, B, and O columns, **b,** oxygen octahedral tilting distributions, **c,d,** Octahedral tilting angle distribution along X and Y directions.

**
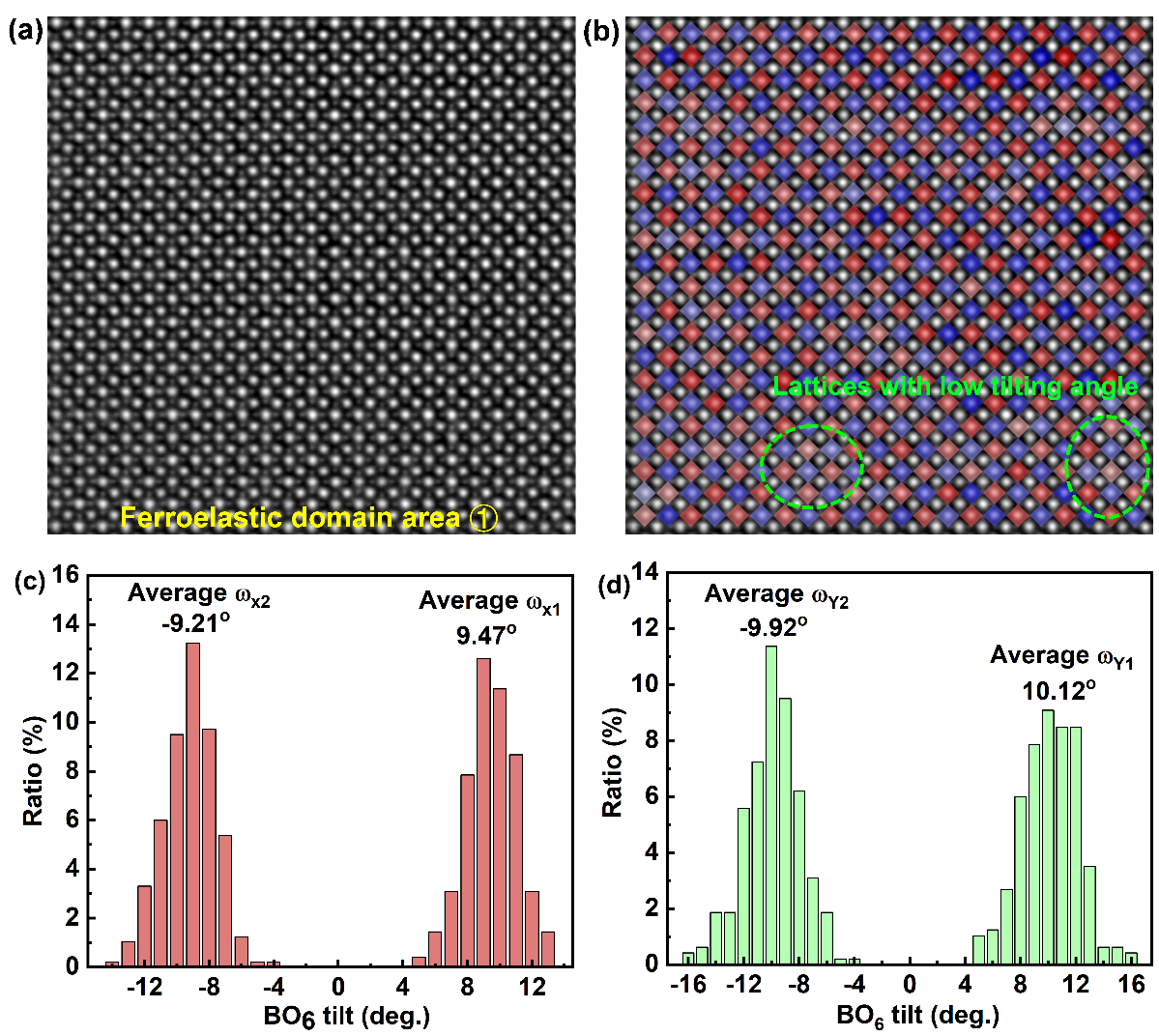
**

Within the ferroelastic domains, long-range ordering of oxygen octahedral tilting can be commonly observed. At the atomic lattice scale, the majority of lattices exhibit high octahedral tilting angles, corresponding to the *P*bnm phase. Previous XRD analysis and DFT calculations indicate this is primarily attributed to the incorporation of small-sized Ca²⁺ ions. This long-range ordering of large-angle oxygen octahedral tilting induces the formation of ferroelastic domain walls (to minimize elastic strain energy). Notably, due to the high-entropy character of the composition, the lattices with low or negligible oxygen octahedral tilting are also present, which could correspond to the coexistent *P*4bm and *R*3c phases.

**Supplementary Fig. 9 Polarization vector map** **corresponding to Fig. 4a viewed along the [001]_pc_ direction. a,** Distribution of polarization vector, and **b,** Statistic distribution of polarization angle.

**
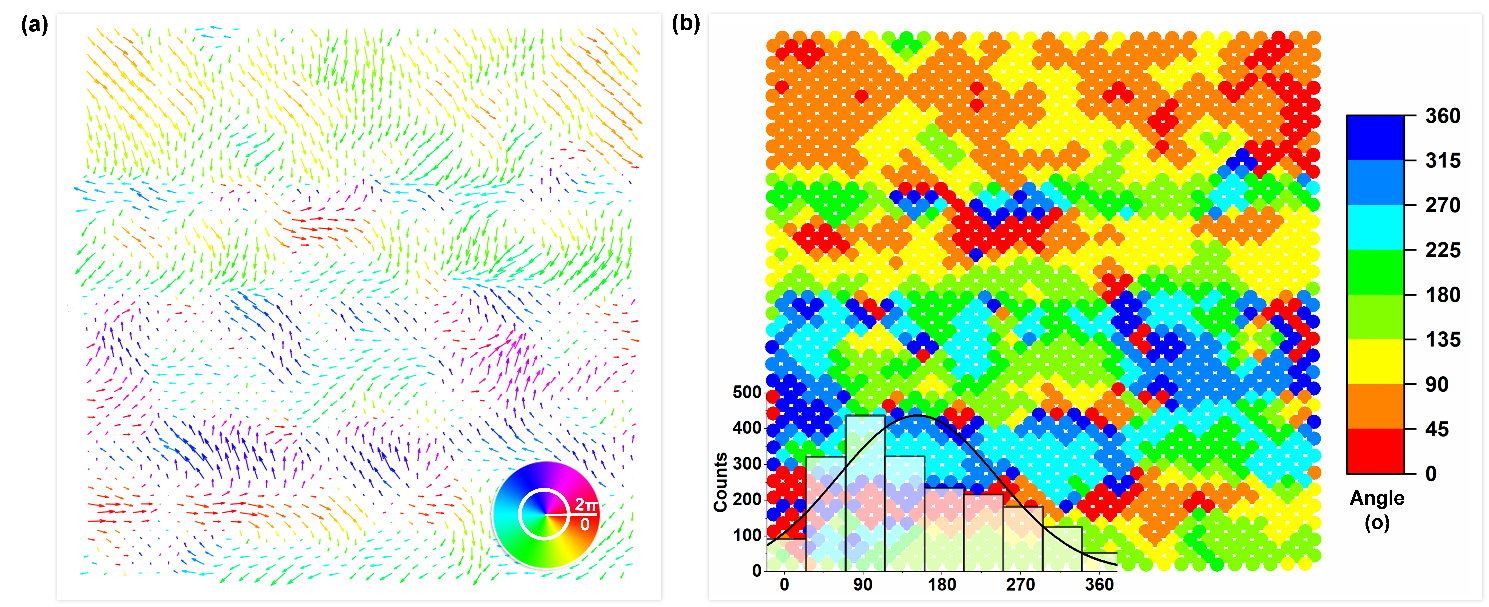
**

**Supplementary Fig. 10 EDS mapping results in the atomic scale for the *x* = 0.22 ceramic.**


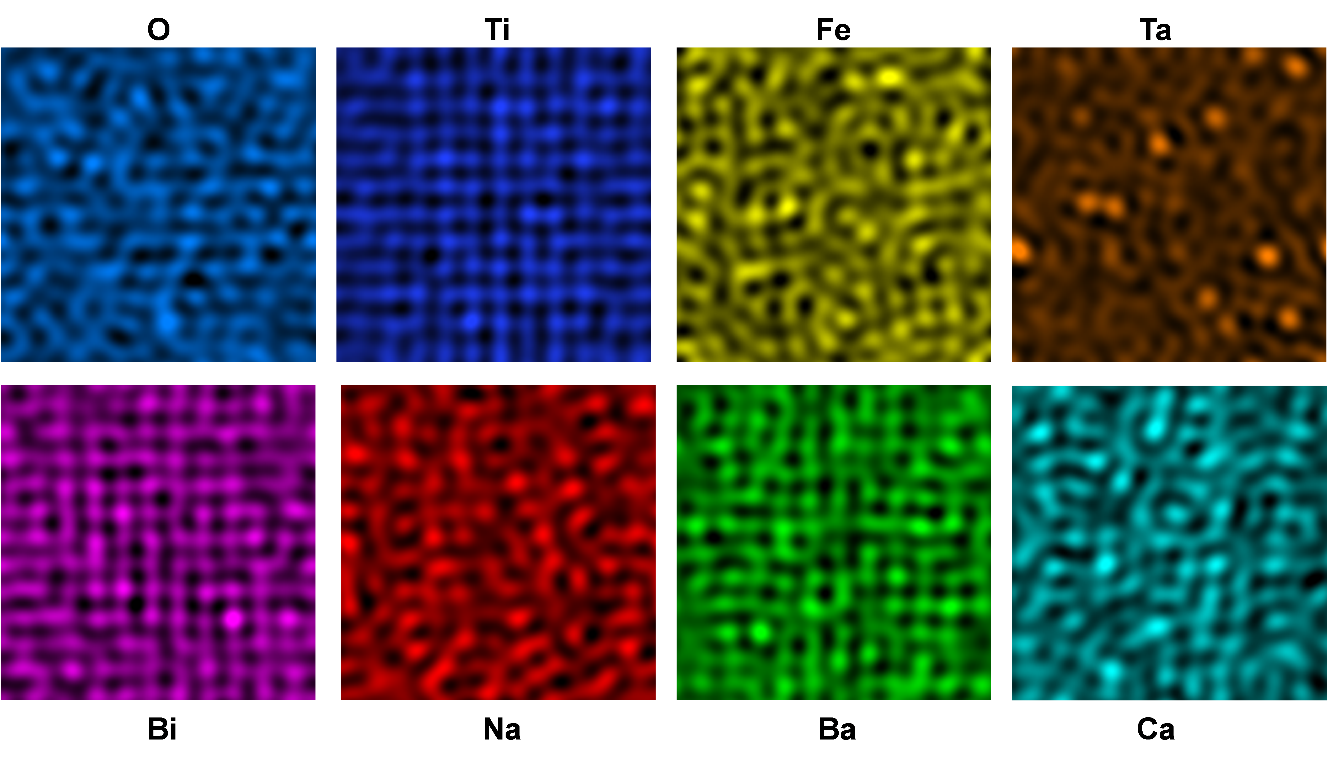


**Supplementary Fig. 11 Analysis of temperature-dependent dielectric spectra** **for the BNBCTFT*_x_* ceramics.** **a, b,** The 1/*ε*_r_-*T* plots of *x* = 0.00 and 0.22 ceramics. **c,** **d,** Nonlinear Vogel-Fulcher fitting of dielectric spectra of *x* = 0.00 and 0.22 ceramics. **e,** **f,** Plots of ln (1/*ε*_r_-1/*ε*_m_) *versus* ln (*T*-*T*_m_) for the *x* = 0.00 and 0.22 ceramics.





Based on the 1/*ε*_r_-*T* plots, it can be found that there are two dielectric anomalies at *T*_S_ and *T*_m_ for the *x* = 0.00 ceramics, which are usually attributed to the thermal evolution of *R*3c and *P*4bm PNRs and the change in size and dynamics of PNRs^[[2](#_ENREF_2)]^. For the *x* = 0.22 ceramic, only one dielectric anomaly observed at *T*_m_ demonstrates the enhanced dispersion of phase transition. Besides, *T*_B_, the Burns temperature, representing the temperature at which polar nanoregions (PNRs) appear during the cooling process, was found to significantly decrease from 390 °C for *x* = 0.00 to 136 °C for *x* = 0.22. It reveals the disruption of long-range ferroelectric ordering and the weakening of dipole-dipole interaction with the increase of *x*. The enhanced relaxor behavior also can be analyzed by the Vogel-Fulcher law and modified Curie-Weiss law, which are used to analyze the freezing temperature ($T_{f}$) and degree of diffuseness ($\gamma$), respectively.

Vogel-Fulcher law:

$$f=f_{0}exp[-\frac{E_{a}}{k_{B}(T_{m}-T_{f})}]$$

where $f_{0}$ and $k_{B}$ are the pre-exponential factor and the Boltzmann constant. For relaxor ferroelectrics, randomly-distributed PNRs in the ergodic relaxor state will grow their sizes with decrease in activity during cooling process and finally freeze into a static nonergodic ordered structure at the freezing temperature ($T_{f}$). The fitting results demonstrate that the $T_{f}$ decreases from 37.6 °C for *x* = 0.00 to -58 °C for *x* = 0.22.

Modified Curie-Weiss law:

$$\frac{1}{\varepsilon}-\frac{1}{\varepsilon_{m}}=\frac{{(T-T_{m})}^{\gamma}}{C}$$

C was the Curie-like constant and the value of *γ* (1 ⩽ *γ* ⩽ 2) is the expression of the dielectric relaxation degree in a ferroelectric. Normally, *γ* = 1, described a Curie–Weiss behavior of normal ferroelectrics, while *γ* = 2 was valid for a classical relaxor ferroelectric. An increase in the *γ* from 1.898 for *x* = 0.00 to 1.945 for *x* = 0.22 further confirms the enhancement of dielectric relaxation with the increase of *x*.

**Supplementary Fig. 12 Comparison of *ε*_r_, tan*δ*, and their temperature stability between the *x* = 0.22 ceramic and representative BaTiO_3_-based, niobate-based, and BNT-based ceramics meeting X9R criteria.**





The temperature stability of dielectric capacitors is usually evaluated by the temperature coefficient of capacitance (TCC), and the current advanced commercial X9R dielectric capacitors based on the BaTiO_3_ system present a working temperature range of –55~200 °C with the TCC less than ±15 %. This figure presents the dielectric performance of representative BaTiO_3_-based^[[3-6](#_ENREF_3)]^, niobate-based^[[7](#_ENREF_7)]^, and BNT-based^[[8-10](#_ENREF_8)]^ ceramics meeting X9R criteria. It can be seen that enhancing the temperature stability of *ε*_r_ always comes at the expense of reducing its absolute value, and it is hard to obtain low tan*δ* in both the high-temperature and low-temperature regions. The *x* = 0.22 ceramic show the advantages in achieving the relatively high *ε*_r_ and low tan*δ* simultaneously over a wide temperature range.

**Supplementary Fig. 13 DC bias-dependent tan*δ* measured at -40~200 °C for the *x* = 0.22 ceramic.**


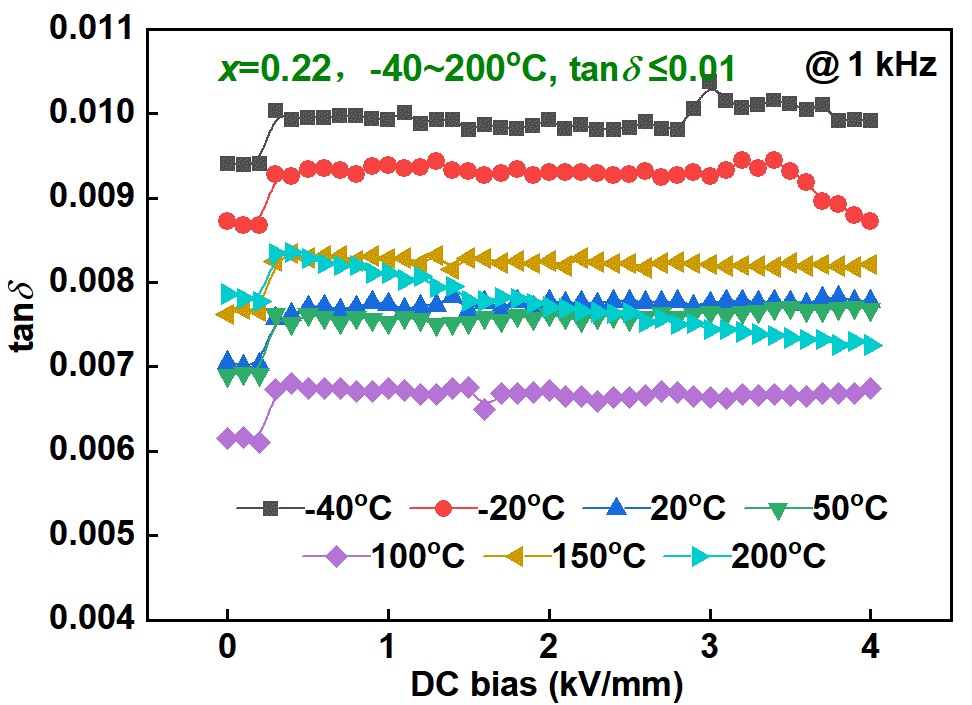


**Fig. 5c** exhibits the excellent DC-bias stability of *ε*_r_ for the *x* = 0.22 ceramic, showing the *α* of only ~2% under a DC bias field of 4 kV/mm and over the wide temperature range of -40~200°C. In general, the *ε*_r_ of ferroelectric ceramics decreases with increasing the DC bias field due to the field-induced domain switching. To be specific, the randomly-distributed ferroelectric domains are triggered to uniformly align along the direction of DC bias, accompanied by the merging and growth of domains. The movement of internal dipoles are inhibited under the effect of DC bias, which reduces the rate of polarization change by applying the AC field, *i.e.* dielectric response. For commercial ceramic capacitors, the value of *α* could reach 20~50% with a DC bias field of 4 kV/mm. However, the *x* = 0.22 ceramic owns the characteristics of ergodic relaxor ferroelectrics within -40~200 °C (*T*_f_~-58°C), accompanying with the polymorphic PNRs. In such a scenario, spontaneous polarization remains confined within PNRs, which stochastically reorients under dominant thermal perturbations. Even under a 4 kV/mm DC bias field, it is hard to establish macroscopic long-range ferroelectric order, which ensures an efficient polarization reversal rate upon AC field application, thereby simultaneously enabling relatively high *ε*_r_ and excellent bias-field stability. On the other hand, within the temperature range of -40~200 °C and DC-bias range of 0~4 kV/mm, the tan*δ* is below 0.01. The increased tan*δ* with decreasing temperature from 100 °C to -40 °C can be attributed to increased polarization loss due to increased size and enhanced interaction of PNRs in the ceramic. The increased tan*δ* at elevated temperatures (e.g. 150 °C and 200 °C) is related to the thermally-activated conduction loss.

**Supplementary Fig. 14 Rietveld refinement of XRD pattern for the *x* = 0.22 ceramic measured at 200 °C.**





**Supplementary Fig. 15 Temperature-dependent Raman spectra of the *x* = 0.22 ceramic. a,** Raman spectra measured at -100~200 °C and the peak fitting results. b, Changes of B-O and BO₆ vibrational modes position with temperature.

**

**

The Raman modes sensitive to BO₆ octahedral motions (e.g., tilting, rotation) shift progressively to lower wavenumbers with increasing temperature, which can be primarily attributed to thermal lattice expansion, reflecting an overall structural softening and a trend towards higher symmetry. Conversely, the Raman modes corresponding to B-O bond stretching vibrations exhibit a distinct blueshift (shift to higher wavenumbers). This contrasting behavior is attributed to the local bond length alterations related to the weakening of ferroelectric distortion.

**Supplementary Fig. 16 Surface SEM micrographs and average grain size *G*_a_ of the thermally-etched BNBCTFT*_x_* ceramics.** **a,** *x* = 0. **b,** *x* = 0.10. **c,** *x* = 0.15. **d,** *x* = 0.22.


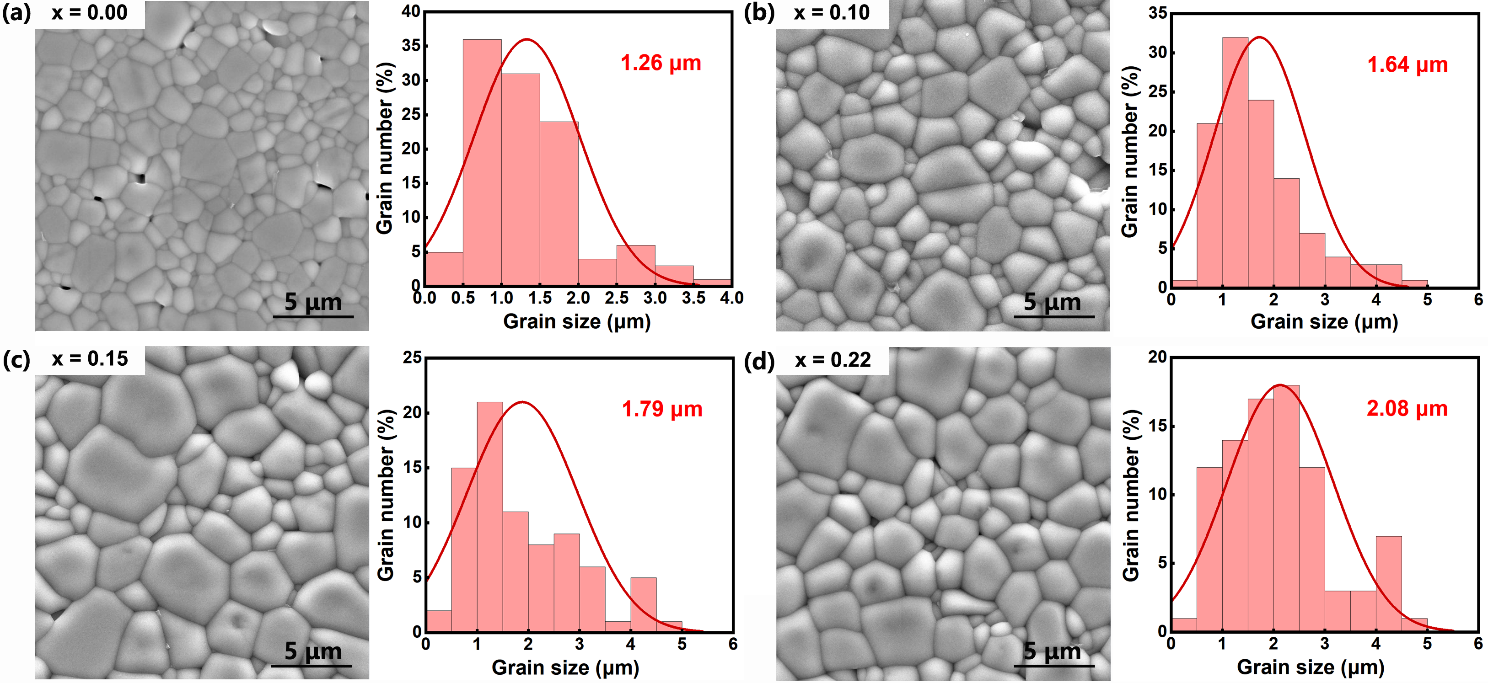


From the SEM images, it can be observed that all the ceramics were well sintered with dense microstructures. The grain size distribution is determined by the linear intercept method, and the results show an increased *G*_a_ from 1.26 μm to 2.08 μm with the increase of *x*. The increase of *G*_a_ could be associated with the variation of the defect structure in BNBCTFT*_x_* ceramics by the introduction of Fe^3+/2+^ ions, which promotes the generation of oxygen vacancies and mass transport during the sintering process.

**Supplementary Fig. 17 Room-temperature *S*-*E* curves for the *x* = 0, 0.10, and 0.22 ceramics.**

**
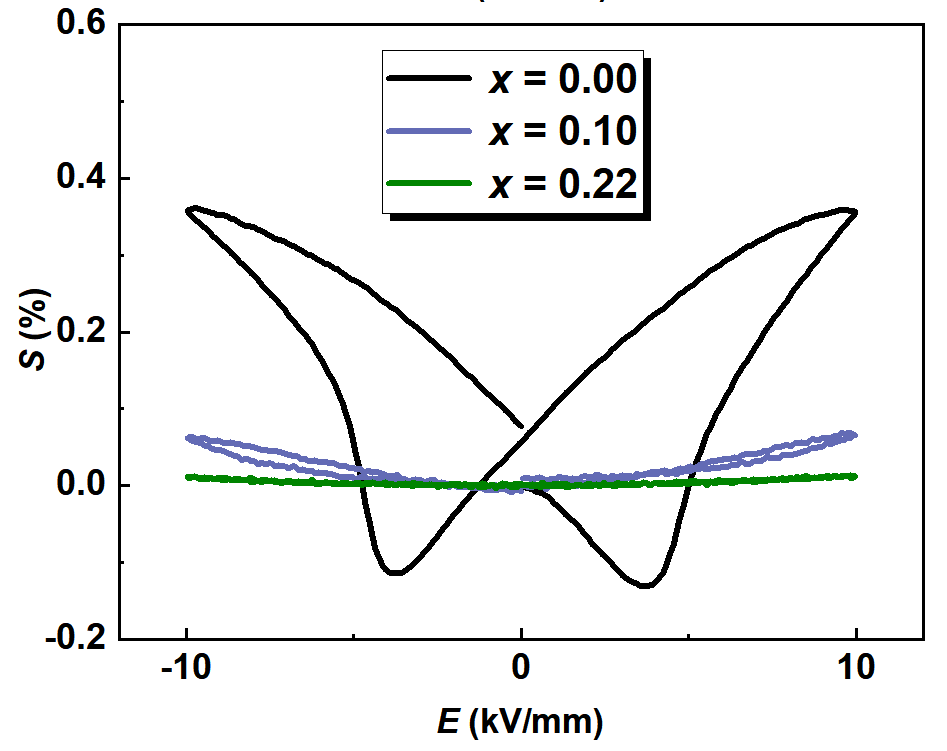
**

**Supplementary Fig. 18** ***In-situ* temperature dependent DC resistivity for the *x* = 0, 0.10, and 0.22 ceramics.**

**
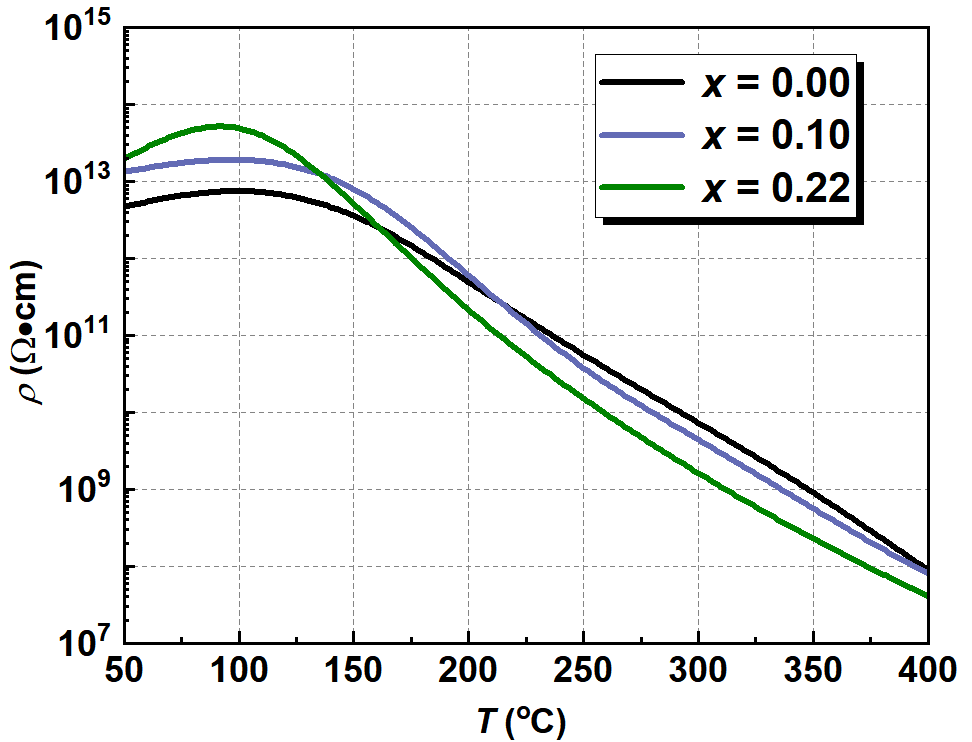
**

**Supplementary Fig. 19 High-resolution O1s XPS spectra of the BNBCTFT*_x_* ceramics a, *x* = 0. b, *x* = 0.10. c, *x* = 0.15. d, *x* = 0.22.**

**
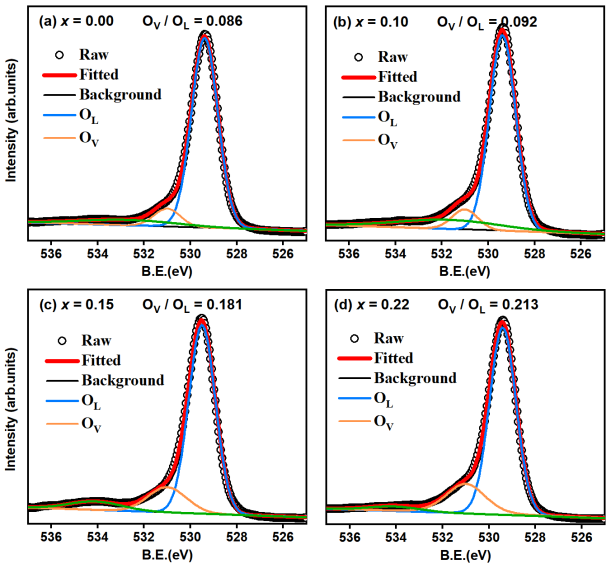
**

The O1s XPS spectra of all samples show the asymmetry and can be consistently fitted by nearly Gaussian curves. The low binding energy centered at ~529.5 eV corresponds to the lattice oxygen (O_L_) including the A–O and B–O bonding, while the binding energy located at ~531 eV is often attributed to the adsorbed oxygen species in oxygen deficient (V_O_) regions. The monotonic decrease in *ρ*_DC_ with temperature for the BNBCTFT*_x_* ceramics shown in **Supplementary Fig. 18** can be ascribed to high-temperature leakage conduction owing to the thermally-activated charge carriers (e.g., oxygen vacancy). The lowest high-temperature *ρ*_DC_ for *x* = 0.22 implies its highest thermally-activated oxygen vacancy concentration, indicated by the XPS analysis of O1s.

**Supplementary Fig. 20 High-resolution Fe 2p XPS spectra of the BNBCTFT*_x_* ceramics. a, *x* = 0.10. b, *x* = 0.15. c, *x* = 0.22.**

**
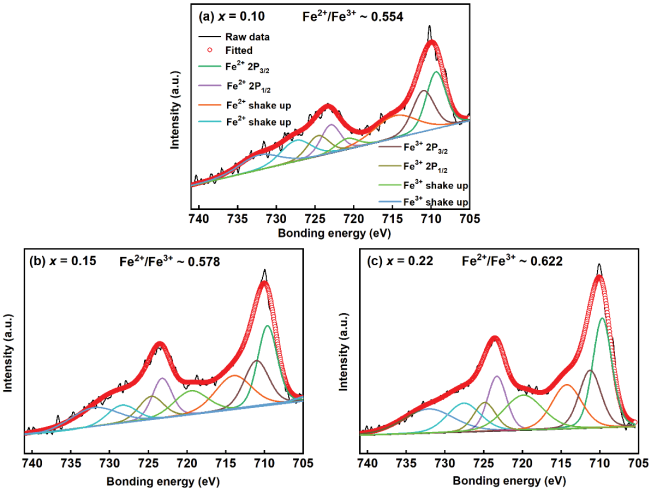
**

Due to the spin-orbit coupling effect, the spectrum of Fe 2p split into doublet peaks of Fe 2p_1/2_ and 2p_3/2_ by 13.6 eV, with the area of Fe 2p_3/2_ twice than that of Fe 2p_1/2_. These peaks are also deconvoluted into Fe^3+^ and Fe^2+^ to determine the concentration of each chemical state of Fe ions. The positions of Fe^3+^ 2p_3/2_ and Fe^2+^ 2p_3/2_ peaks are located at ~710.9 and ~709.6 eV, respectively. The XPS analysis of Fe 2p demonstrates the valence state change of Fe^3+^ to Fe^2+^ in the BNBCTFT*_x_* ceramics and increased Fe^2+^/Fe^3+^ ratio with the increase of *x*, which induce the formation of oxygen vacancies for charge compensation.

**Supplementary Fig. 21 Frequency stability of dielectric energy storage properties for the *x* = 0.22 ceramic. a,** Frequency-dependent *ε*_r_ and tan*δ* measured at different temperatures. **b,** Frequency-dependent *P*-*E* loops, *W*_rec_, and *η* measured at 40 kV/mm and room-temperature.

**
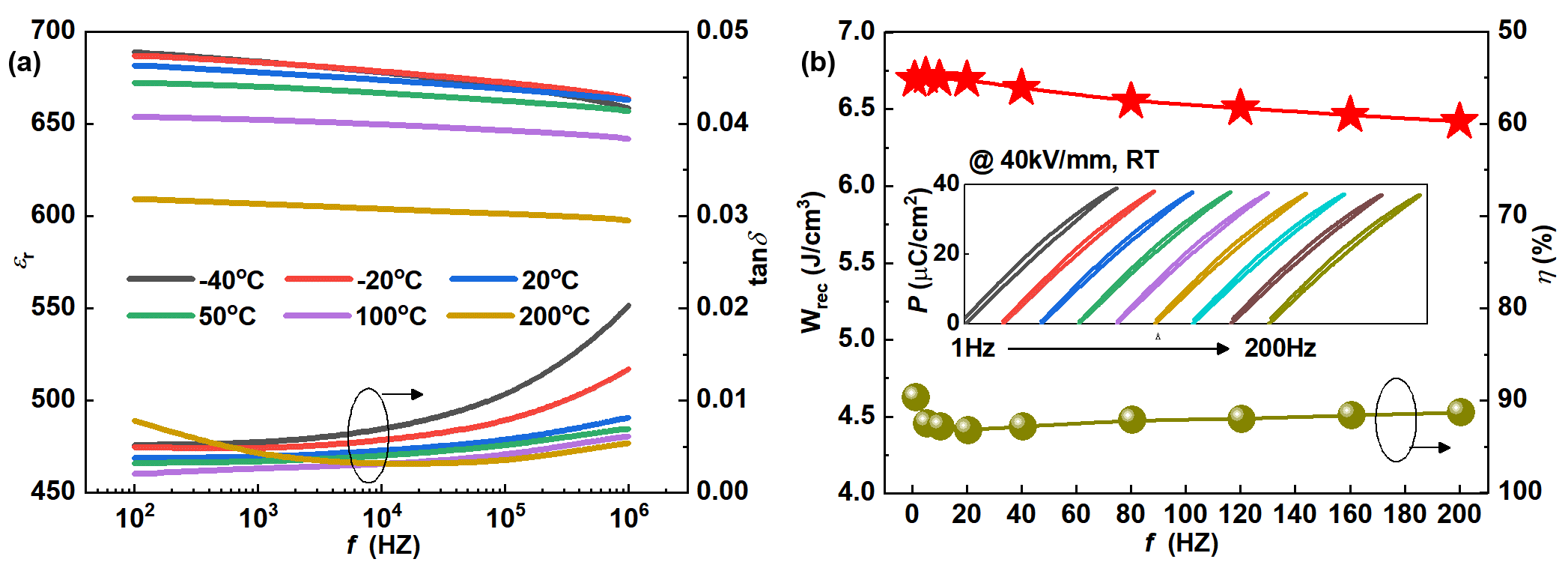
**

**Supplementary Table 1** **Refined lattice parameters by the Rietveld method for the BNBCTFT*x* ceramics measured at room temperature.**

| *x* | Space group | Weight fraction (%) | Lattice parameters | V(Å^3^) |
| --- | --- | --- | --- | --- |
| 0.00 | *P*4bm | 97.7 | a=b=5.5300(9) Å, c=3.9107(6) Å  α=β=γ=90^o^ | 119.59(9) |
|  | *R*3c | 2.3 | a=b=5.5158 (3) Å, c=13.6536(8) Å  α=β=90^o^, γ=120 ^o^ | 359.75(1) |
| 0.10 | *P*4bm | 86.8 | a=b=5.5161(7) Å, c=3.9005(7) Å  α=β=γ=90^o^ | 118.68(7) |
|  | *R*3c | 0.4 | a=b=5.5234(8) Å, c=13.5712(8) Å  α=β=90^o^, γ=120 ^o^ | 358.57(2) |
|  | *P*bnm | 12.8 | a=5.5317(3) Å, b=5.5157(9) Å, c=7.8446(4)Å  α=β=γ=90^o^ | 239.35(4) |
| 0.15 | *P*4bm | 72.2 | a=b=5.5127(8) Å, c=3.8981(2) Å  α=β=γ=90^o^ | 118.46(7) |
|  | *R*3c | 1.3 | a=b=5.4892(3) Å, c=13.6296(4) Å  α=β=90^o^, γ=120 ^o^ | 355.66(2) |
|  | *P*bnm | 26.5 | a=5.5318(6) Å, b=5.5097(9) Å, c=7.8092(2) Å  α=β=γ=90^o^ | 238.02(2) |
| 0.22 | *P*4bm | 23.6 | a=b=5.5063(3) Å, c=3.8938(0) Å  α=β=γ=90^o^ | 118.05(9) |
|  | *R*3c | 3.2 | a=b=5.4917(1) Å, c=13.5902 (2) Å  α=β=90^o^, γ=120 ^o^ | 354.95(4) |
|  | *P*bnm | 73.2 | a=5.5070(7)Å, b=5.5227(0) Å, c=7.7864(1) Å  α=β=γ=90^o^ | 236.81(6) |

**Reference**

[1] Sarkar, A.; Wang, Q.; Schiele, A.; Chellali, M. R.; Bhattacharya, S. S.; Wang, D.; Brezesinski, T.; Hahn, H.; Velasco, L.; Breitung, B. High-Entropy Oxides: High-Entropy Oxides: Fundamental Aspects and Electrochemical Properties. *Advanced Materials* **31**, 1970189 (2019).

[2] Jo, W.; Schaab, S.; Sapper, E.; Schmitt, L. A.; Kleebe, H.-J.; Bell, A. J.; Rödel, J. On the phase identity and its thermal evolution of lead free (Bi_1/2_Na_1/2_)TiO_3_-6mol%BaTiO_3_. *J. Appl. Phys.* **110**, 074106 (2011).

[3] Muhammad, R.; Ali, A.; Camargo, J.; Castro, M.; Lei, W.; Song, K.; Wang, D. Enhanced Thermal Stability in Dielectric Properties of NaNbO_3_-Modified BaTiO_3_–BiMg_1/2_Ti_1/2_O_3_ Ceramics for X9R-MLCC Applications. *Crystals* **12**, 141 (2022).

[4] Sun, Y.; Liu, H.; Hao, H.; Zhang, L.; Zhang, S. The role of Co in the BaTiO_3_–Na_0.5_Bi_0.5_TiO_3_ based X9R ceramics. *Ceramics International* **41**, 931-939 (2015).

[5] Zhang, W.; Yang, J.; Wang, F.; Chen, X.; Mao, H. Enhanced dielectric properties of La-doped 0.75BaTiO3-0.25Bi(Mg0.5Ti0.5)O3 ceramics for X9R-MLCC application. *Ceramics International* **47**, 4486-4492 (2021).

[6] Jiang, X.; Hao, H.; Yang, Y.; Zhou, E.; Zhang, S.; Wei, P.; Cao, M.; Yao, Z.; Liu, H. Structure and enhanced dielectric temperature stability of BaTiO_3_-based ceramics by Ca ion B site-doping. *Journal of Materiomics* **7**, 295-301 (2021).

[7] Chen, L.; Zhang, Y.; Qi, H.; Wang, R.; Ji, Y.; Nishikubo, T.; Azuma, M.; Zhou, C.; Chen, J. Ultrahigh Capacitive Energy Storage in Lead-Free Relaxors via Localizing Distortion. *ACS nano* **19**, 17738-17745 (2025).

[8] Jia, W.; Hou, Y.; Zheng, M.; Xu, Y.; Yu, X.; Zhu, M.; Yang, K.; Cheng, H.; Sun, S.; Xing, J. Superior temperature-stable dielectrics for MLCCs based on Bi_0.5_Na_0.5_TiO_3_-NaNbO_3_ system modified by CaZrO_3_. *Journal of the American Ceramic Society* **101**, 3468-3479 (2018).

[9] Zhou, X.; Qi, H.; Yan, Z.; Xue, G.; Luo, H.; Zhang, D. Superior thermal stability of high energy density and power density in domain-engineered Bi_0.5_Na_0.5_TiO_3_-NaTaO_3_ relaxor ferroelectrics. *ACS Applied Materials & Interfaces* **11**, 43107-43115 (2019).

[10] Schulz, T.; Veerapandiyan, V.; Deluca, M.; Töpfer, J. Synthesis and properties of lead-free BNT-BT-xCZ ceramics as high-temperature dielectrics. *Materials Research Bulletin* **145**, 111560 (2022).
